# Supplementary material for: Lhcx proteins provide photoprotection via thermal dissipation of absorbed light in the diatom Phaeodactylum tricornutum
Source: Nat Commun. 2019 Sep 13;10:4167. doi: 10.1038/s41467-019-12043-6 (PMC6744471; doi:10.1038/s41467-019-12043-6)
Supplement: Supplementary file 1 — Supplementary Information [file 41467_2019_12043_MOESM1_ESM.pdf]

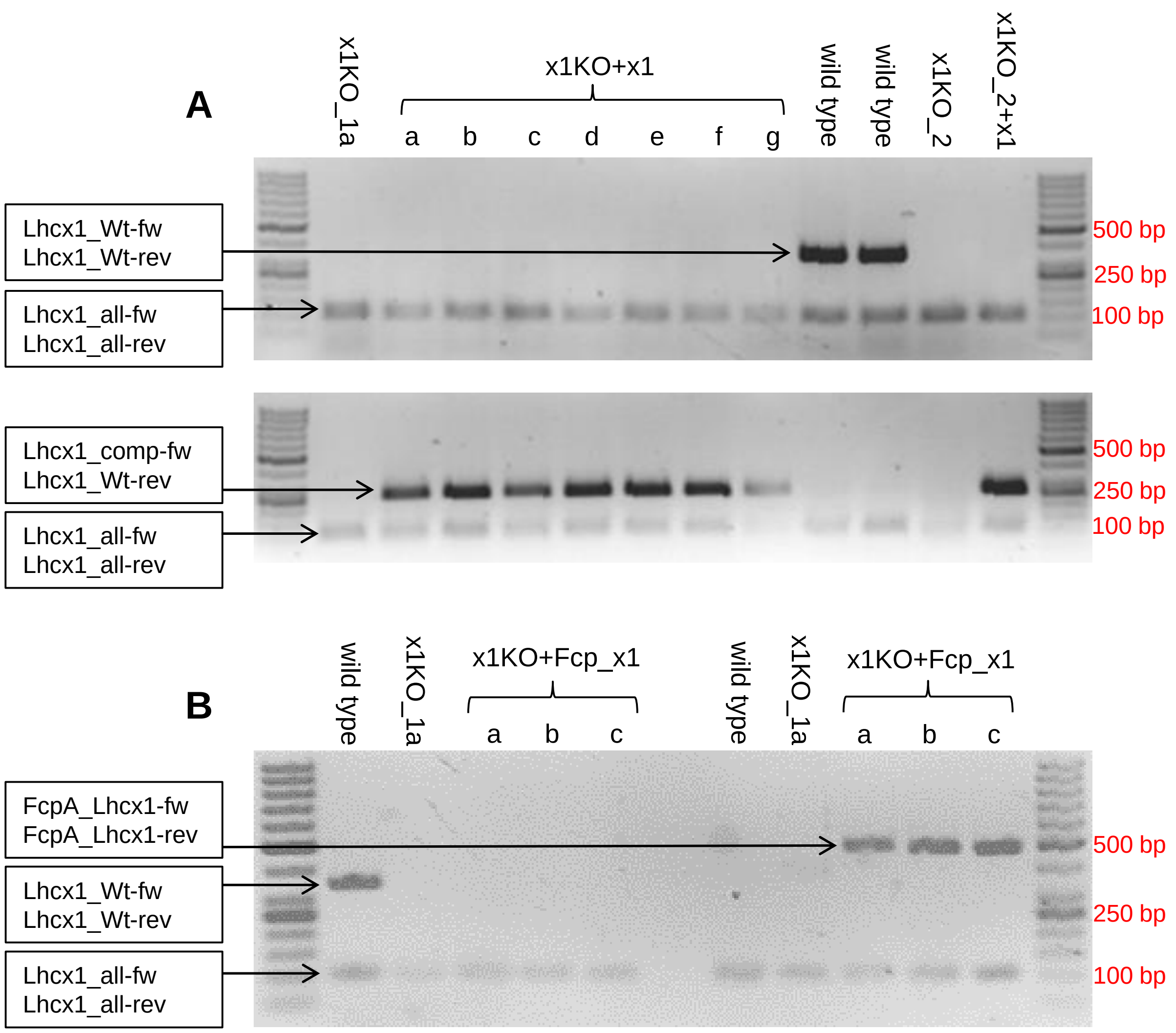

Supplementary Figure 1: Screening of *P. tricornutum* x1KO and complemented lines via PCR

A) Strains are characterized where the x1KO_1a/2 was complemented with an *Lhcx1* gene under the control of the *Lhcx1* promoter and terminator. B) Strains are characterized where x1KO was complemented with an *Lhcx1* gene under the control of an *FcpA* promoter and terminator.

The primer combination Lhcx1_all-fw and Lhcx1_all-rev amplifies 102 bp at the 3'-end of the *Lhcx1* gene which is still intact in the x1KO lines. The primer combination Lhcx1_Wt-fw and Lhcx1_Wt-rev amplifies a 347 bp fragment only in the Wt *Lhcx1* gene but not in the x1KO lines, despite spanning the target sites for both TALEN pairs used to produce the x1KO lines. Using the primer combination Lhcx1_comp-fw/Lhcx1_Wt-rev yields a product of 262 bp, which is only present in the complemented x1KO+x1 lines, where an *Lhcx1* gene without introns and with synonymous codon exchanges of the TALEN binding sites had been introduced under the control of the *Lhcx1* promoter and terminator. The primer combination FcpA_Lhcx1-fw and FcpA_Lhcx1-rev amplifies a 479 bp fragment, which includes the whole *FcpA* promoter and the first part of the modified *Lhcx1*. FcpA_Lhcx1-rev primer is only able to bind to the first modified TALEN site of the complementation construct, while it cannot bind to the natural *Lhcx1* gene. In B, the PCR for the five DNA samples on the left had been performed with the primer combination Lhcx1_Wt-fw/rev, while for those five samples on the right the primer combination FcpA_Lhcx1-fw/rev was used. The size of the corresponding marker bands is indicated in red. Source data are provided as a Source Data file.

x1KO_1a

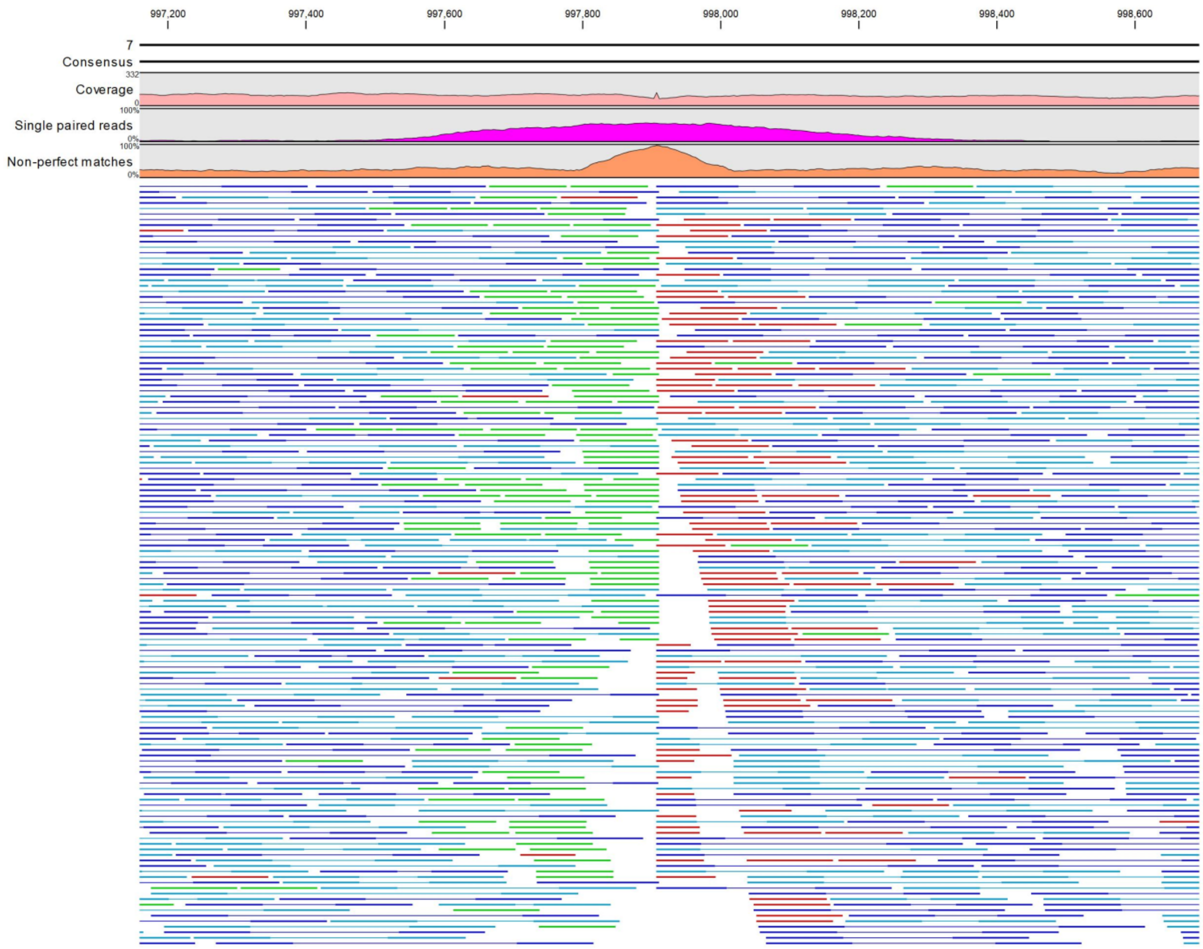
997,200
997,400
997,600
997,800
998,000
998,200
998,400
998,600
7
Consensus
Coverage
Single paired reads
Non-perfect matches

x1KO_1b

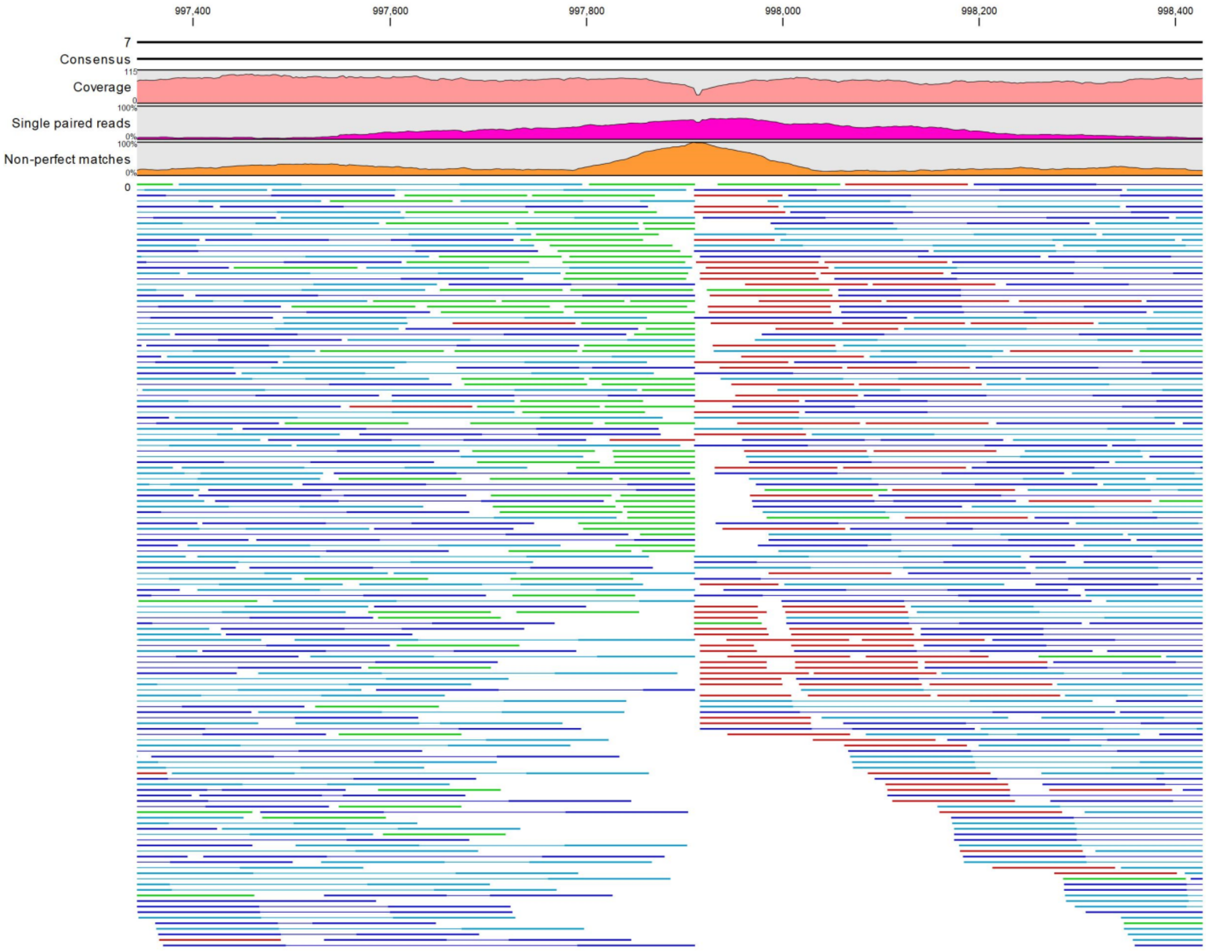
997,400
997,600
997,800
998,000
998,200
998,400
7
Consensus
Coverage
Single paired reads
Non-perfect matches
0

x1KO_2

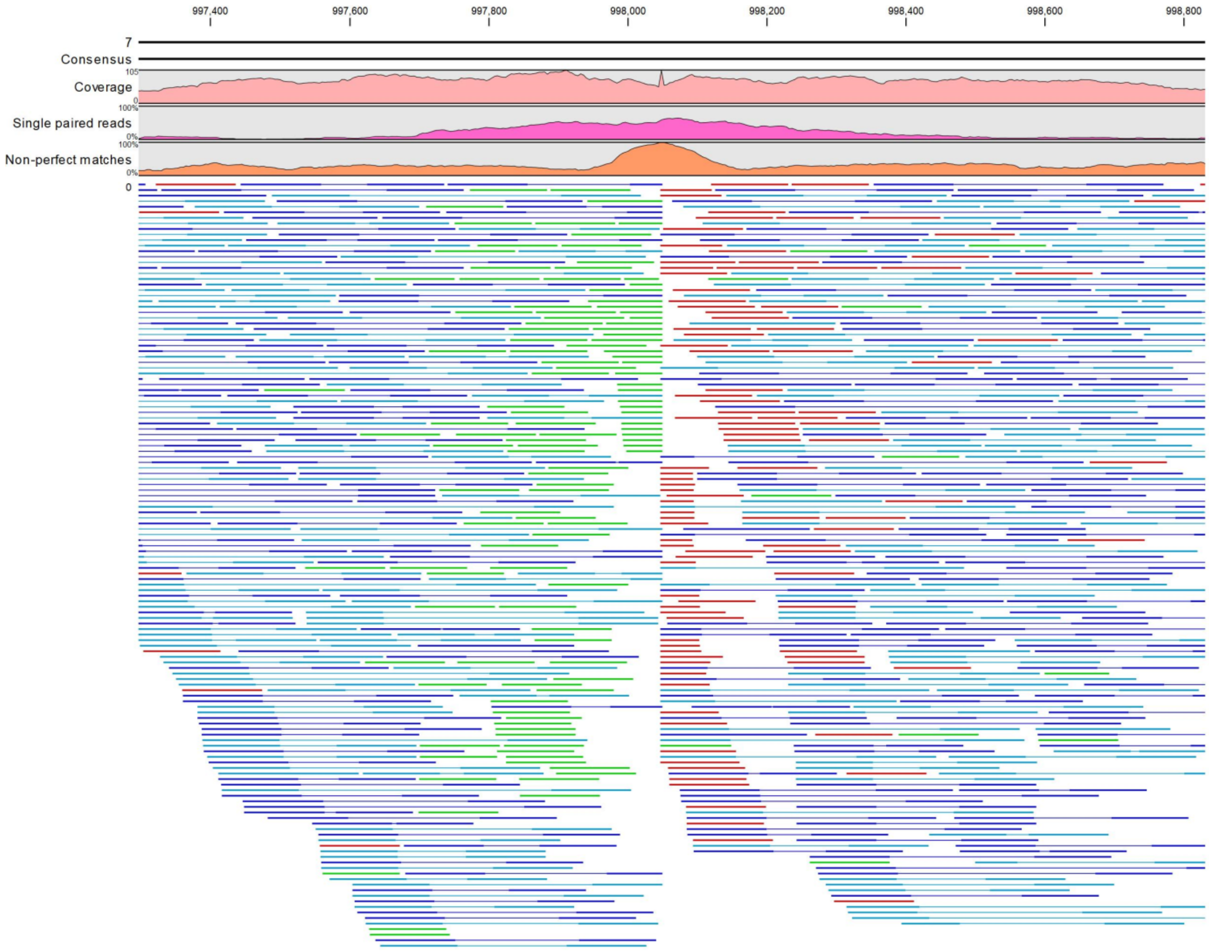

Supplementary Figure 2: DNA sequencing results of the three *P. tricornutum* x1KO lines in the region of the *Lhcx1* gene

Illumina 125 bp paired end sequencing was performed. We obtained 23.4, 30.9, 20.1, and 12.6 million reads for the Wt4, x1KO_1a, x1KO_1b and x2KO strain, respectively. Graphical illustration was done using using CLC Genomics (Qiagen, Germany).The blue/cyan bars indicate paired reads, with thicker lines depicting the sequenced reads and the thinner lines in between representing the region of the Wt4 (UTEX646) genome to which this part fits. Green bars indicate forward reads where the paired reverse read could not fit to the reference genome of Wt4. Red bars indicate reverse reads where the paired forward read could not be mapped to the reference genome. For all three x1KO lines, the region around the TALEN cutting site (~997900 on chromosome 7 for the TALEN pair 1, ~998050 for TALEN pair 2) was only covered be single reads and never any paired read spanning this region could be observed, likely due to large insertions. This is also supported by the orange labelled non-perfect matches chart, reaching 100% in this region in three x1KO lines. Raw sequencing reads can be obtained from the ENA depository under the accession code "PRJEB33825[https://www.ebi.ac.uk/ena/data/view/PRJEB33825]".

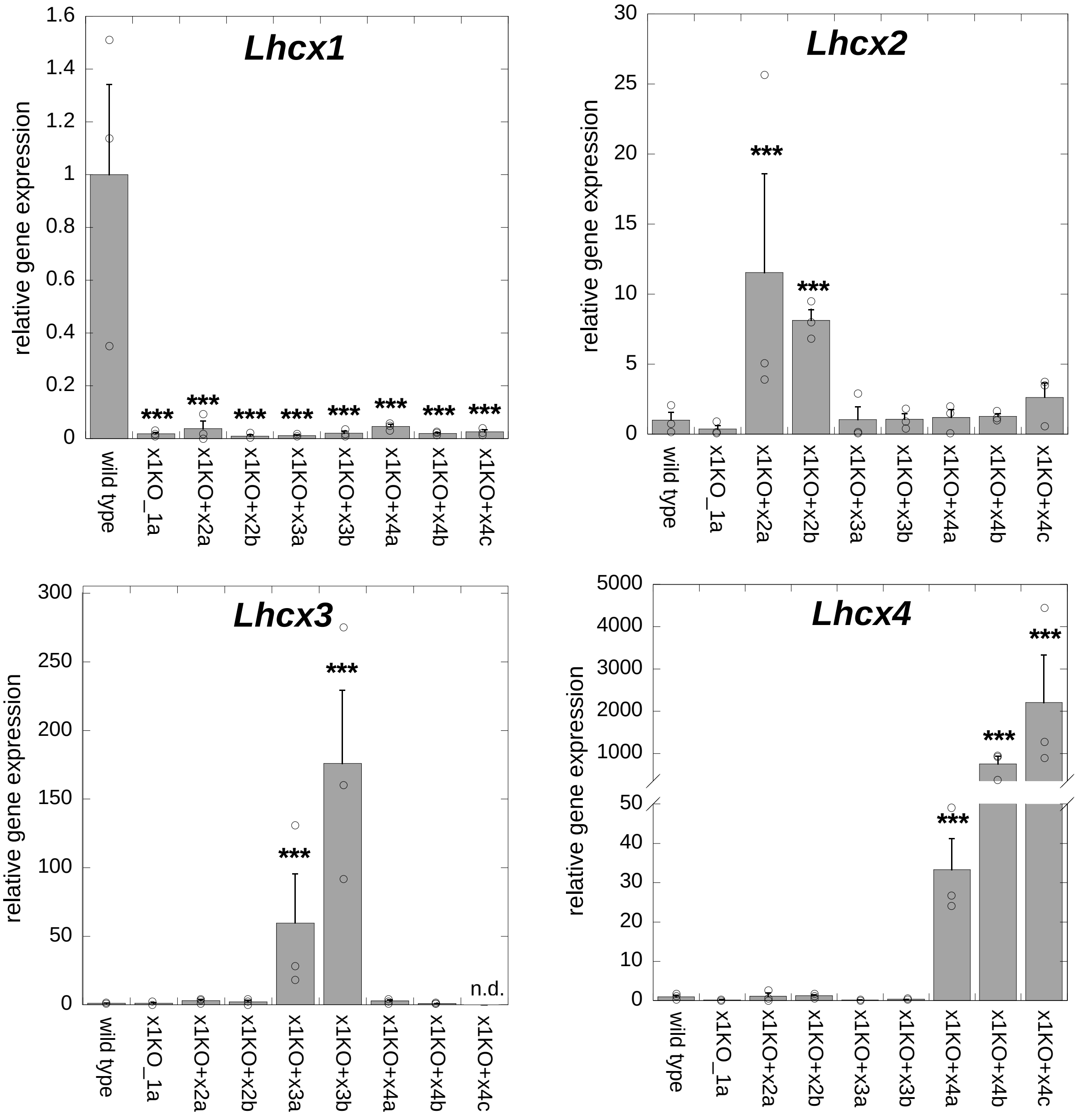

Supplementary Figure 3: *Lhcx* expression of modified *P. tricornutum* strains cultivated under low light

Relative expression was normalized to expression of the *18s* gene, and then gene expression of the mutated strains was normalized to that of the wild type. Three biological replicates, each measured in technical triplicates, were analyzed and the mean + SE is indicated. Mean values of the biological replicates were tested for statistical significance using a randomization test performed by the REST algorithm. *** indicates $p < 0.001$ compared to wild type expression. n.d., not detected. Source data are provided as a Source Data file.

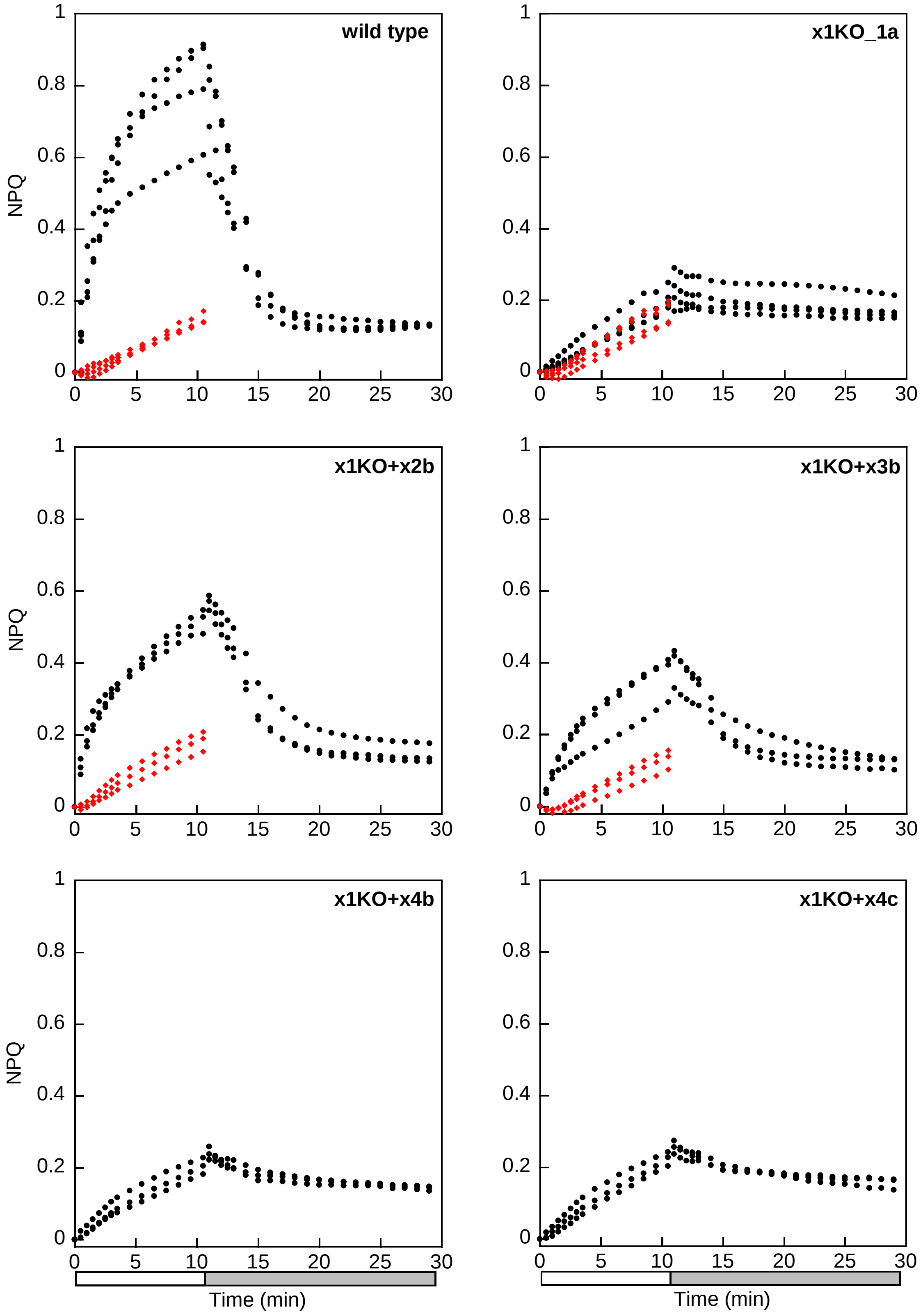

Supplementary Figure 4: NPQ capacity of wild type and mutants

Wild type (4 biological replicates (BR)), x1KO_1a (4 BR), x1KO+x2b (3 BR), x1KO+x3b (3 BR), x1KO+x4b (3 BR) and x1KO+x4c (3 BR) cells were exposed to 1700 µmol photons m^{-2} s^{-1} (10 min, white bar), followed by 18 min of low light (grey bar). Strains were concentrated to 10 mg chlorophyll *a* L^{-1}. Red points indicate samples which had been incubated with DTT prior to high light exposure in order to prevent diatoxanthin formation. Source Data are provided as a Source Data file.

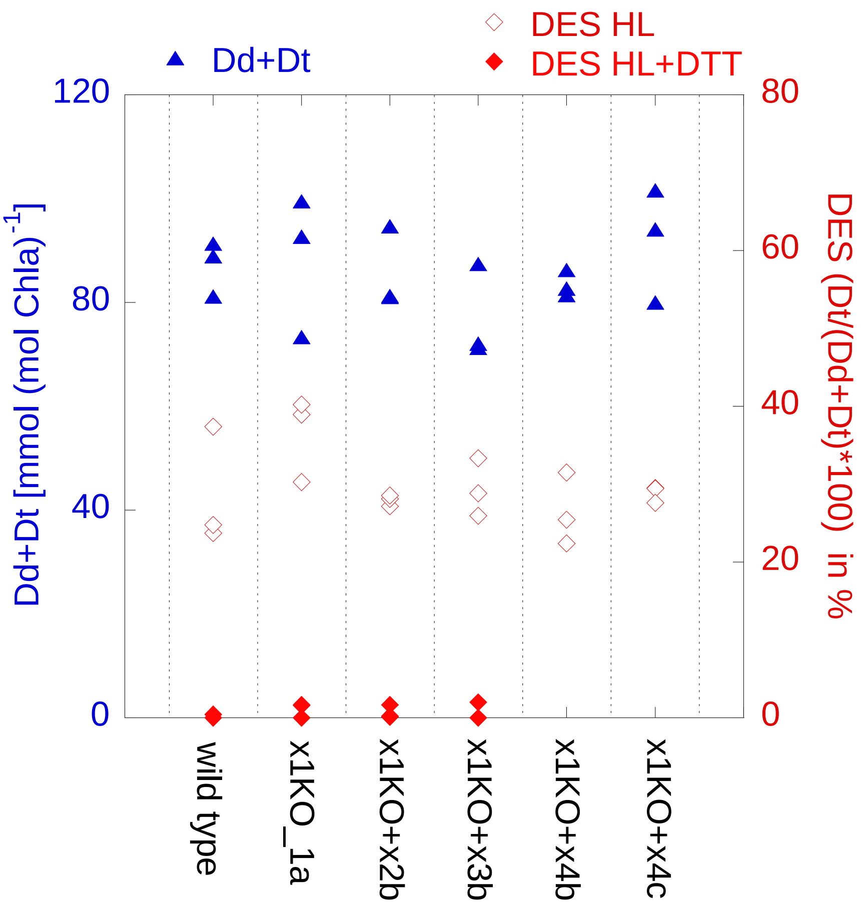

Supplementary Figure 5: Pool size of diadinoxanthin+diatoxanthin (Dd+Dt) and de-epoxidation state (DES)

Cells were exposed to 10 min of 1700 µmol photons m^{-2} s^{-1} without and with prior application of DTT Three biological replicates each were measured. Source data are provided as a Source Data file.

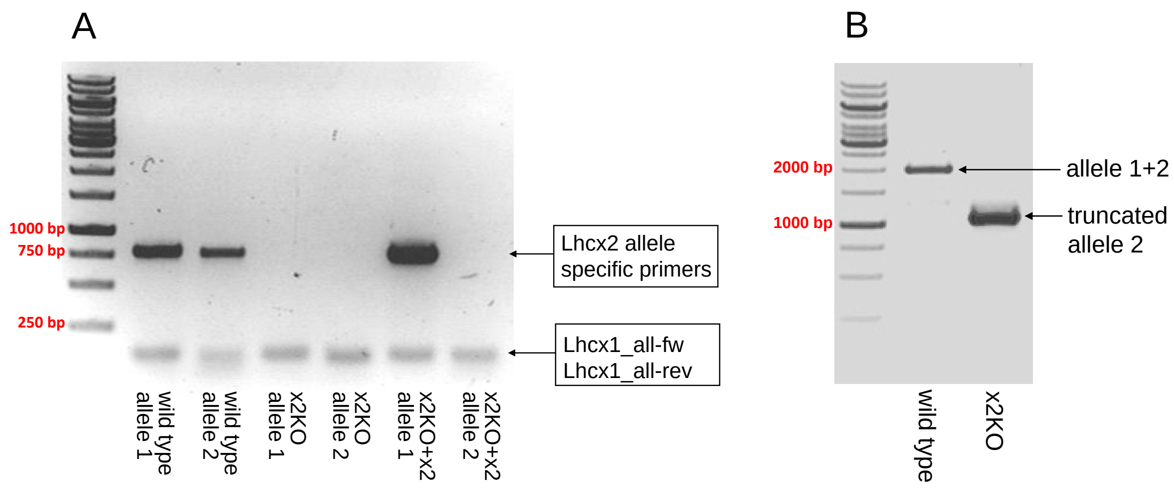

Supplementary Figure 6: Characterization of *P. tricornutum* x2KO and complemented lines via PCR and gel electrophoresis

A) The two *Lhcx2* alleles were amplified via allele specific primers (primer combinations Lhcx2_allele1-fw/Lhcx2_allele1-rev and Lhcx2_allele2-fw/ Lhcx2_allele2-rev, respectively). While in the wild type both alleles could be successfully amplified, they could not be detected in the x2KO strain. In the complemented x2KO+x2 line, the introduced allele 1 was detected. The primer combination Lhcx1_all-fw and Lhcx1_all-rev amplifies 102 bp at the 3'-end of the *Lhcx1* gene, serving as a PCR positive control. B) PCR using the primer combination Lhcx2_prom-fw and Lhcx2_term-rev which spans the entire coding region of the *Lhcx2* gene. A band could be amplified in the x2KO strain which, after Sanger sequencing, was identified as a truncated allele 2 of *Lhcx2*, with a 750 bp deletion of the gene sequence and additional ~170 bp deletion of the terminator. In red, the size of the corresponding marker bands are indicated. Source data are provided as a Source Data file.

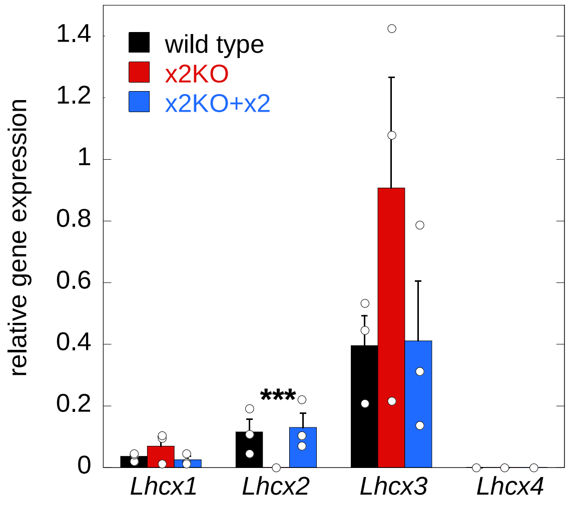

Supplementary Figure 7: *Lhcx* expression of wild type, x2KO and x2KO+x2 upon exposure to 2 hours of ~700 µmol photons m^{-2} s^{-1}

Relative expression was normalized to expression of *18s* gene. Three biological replicates, each measured in technical triplicates, were analyzed and tested for statistical significance using the REST algorithm. *** indicates p<0.001 compared to wild type expression. SE is indicated. A different light intensity compared to Supplementary Figure 9 had been used, because we needed to use another sample setup, as the cuvette in the Dual-PAM did not allow harvesting enough cells for RNA isolation. Source data are provided as a Source Data file.

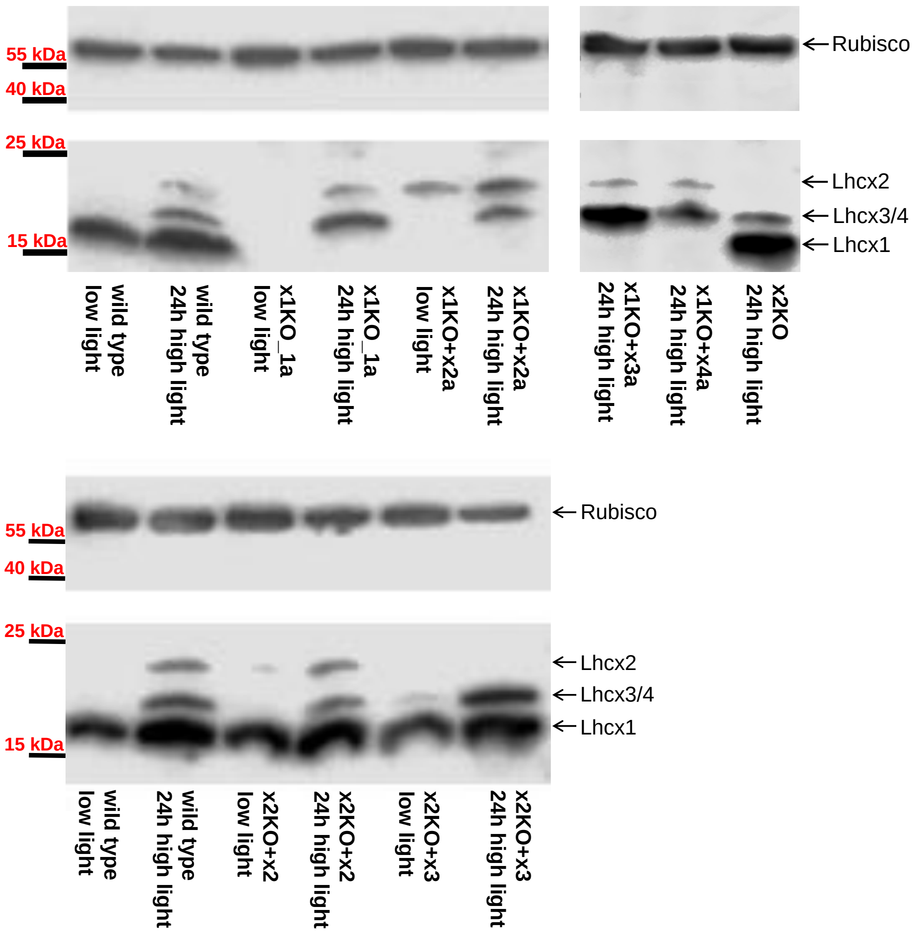

Supplementary Figure 8: Western blots of wild type and mutants from 24 h high light (~400 µmol photons m^{-2} s^{-1}) grown cultures

The upper blots show the wild type, x1KO_1a, the supplemented x1_KO lines and the x2KO line, while the x2KO complemented with Lhcx2 and supplemented with Lhcx3 is shown in the lower blot. For comparison, some low light grown cultures were also analyzed. Three different blots (left, right and bottom) are shown. After blotting, the blots were cut and the upper half was incubated with a Rubisco antibody, while the lower half was incubated with the Lhcx antibody. Lhcx1 has the lowest, Lhcx2 the highest, and Lhcx3 and Lhcx4 have a molecular weight in between. Lhcx2 protein is not detectable under low light cultivation in the wild type, but only after prolonged high light cultivation. The x2KO line does not express Lhcx2 even after high light cultivation (upper right blot) as the x2KO+x3 line does not, too (lower blot). Source data are provided as a Source Data file.

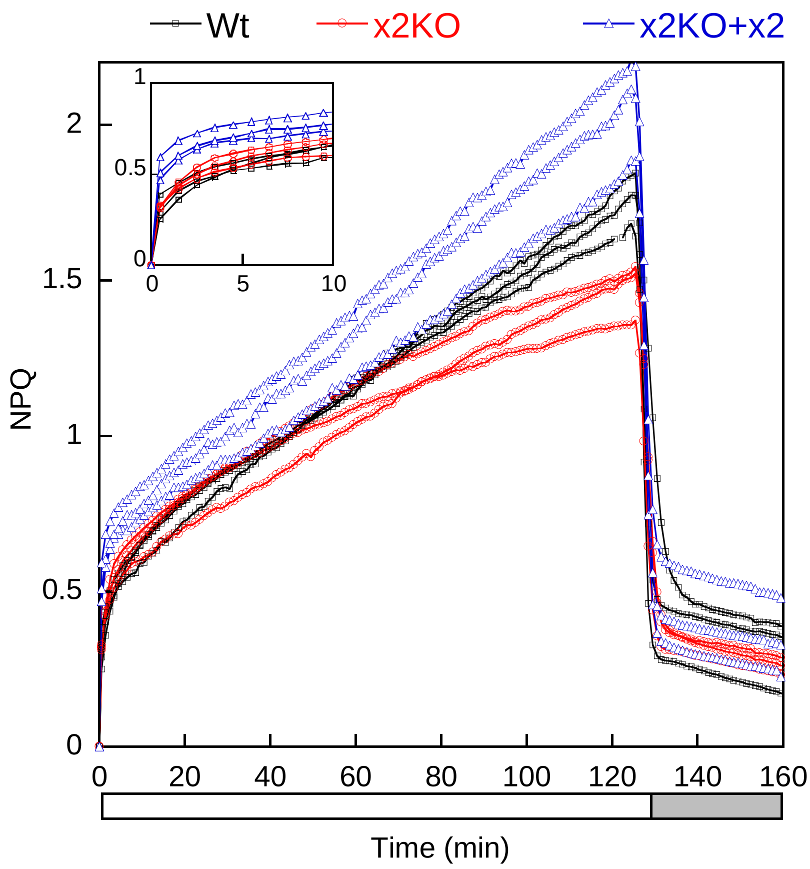

Supplementary Figure 9: NPQ capacity in wild type, x2KO and x2KO+x2 strains upon exposure to 130 min of 1700 µmol photons m^{-2} s^{-1} (white bar), followed by 30 min of low light recovery (grey bar) Three biological replicates are indicated. The inset shows the first 10 min of NPQ development in the three strains. Note that there are no major differences in NPQ capacity between the Wt and the x2KO line during the first hour of illumination, while during the second hour the Wt develops a higher NPQ capacity. The x2KO+x2 has a somewhat higher NPQ capacity right from the beginning. Source data are provided as a Source Data file.

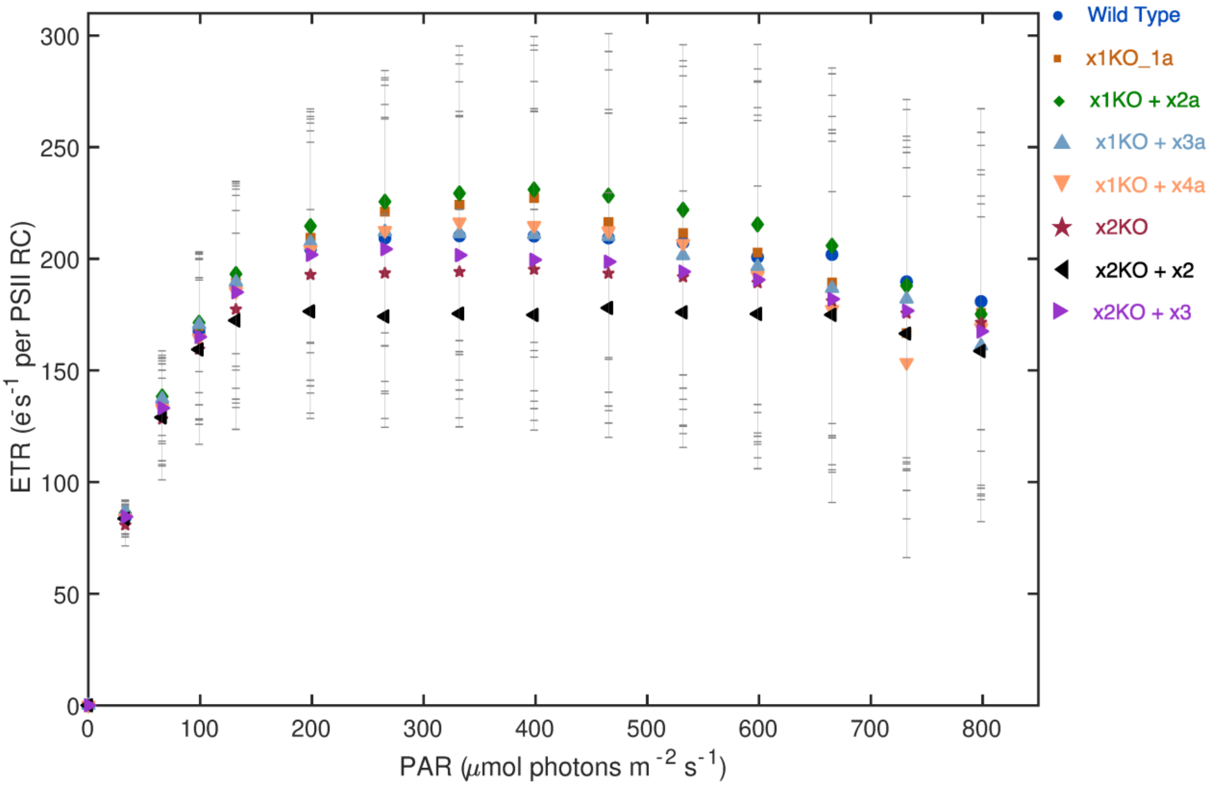

Supplementary Figure 10: Electron transport rates during rapid light curves of Wt and mutants grown under low light

Values are the mean of six biological replicates. SD is given. Source data are provided as a Source Data file.

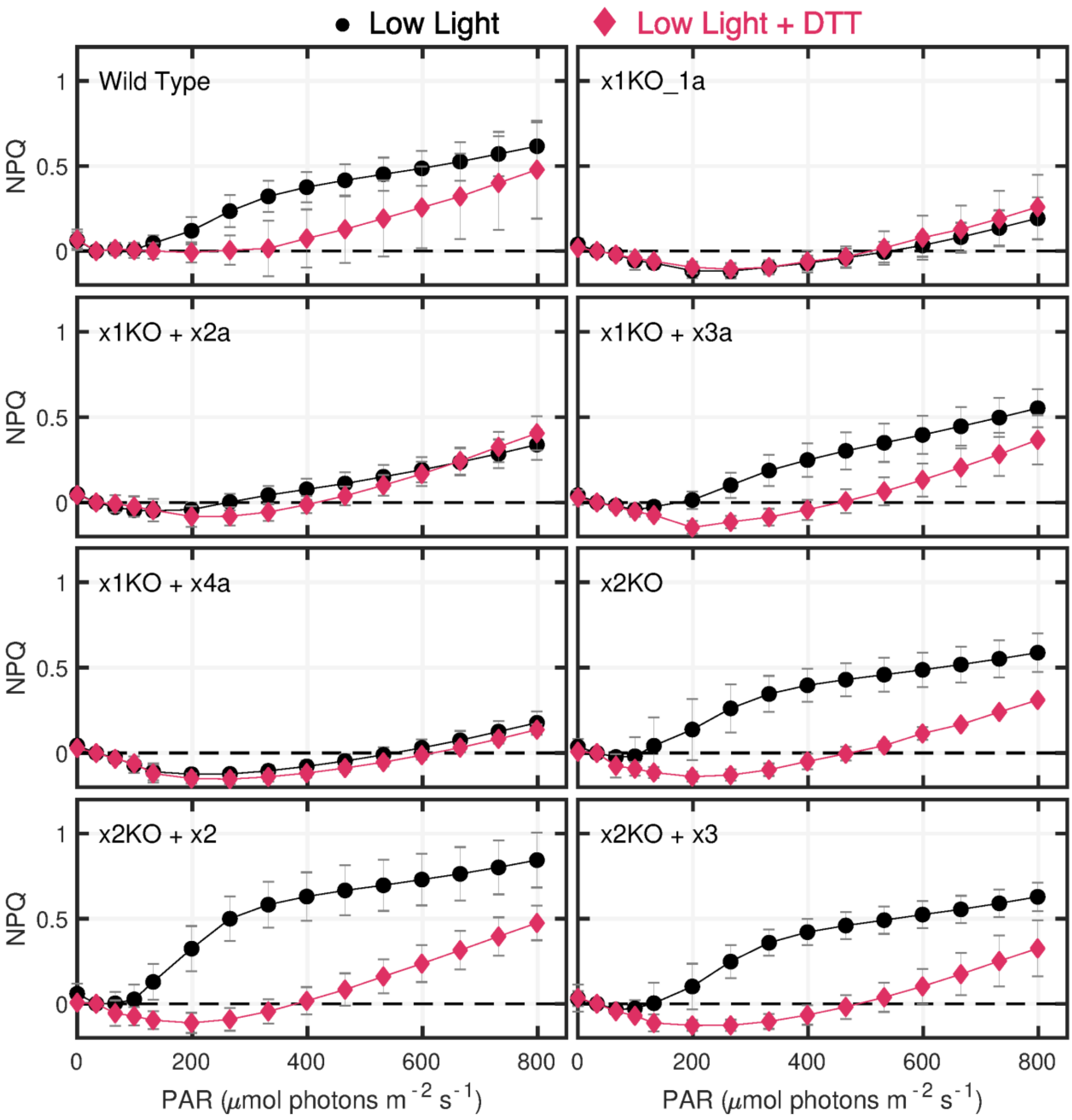

Supplementary Figure 11: NPQ development during rapid light curves of Wt and mutants grown under low light

Samples were exposed to increasing light intensities without or with prior incubation with DTT. Values are the mean of six (-DTT) or three (+DTT) biological replicates. SD is given. Dashed black line denotes zero NPQ. Source data are provided as a Source Data file.

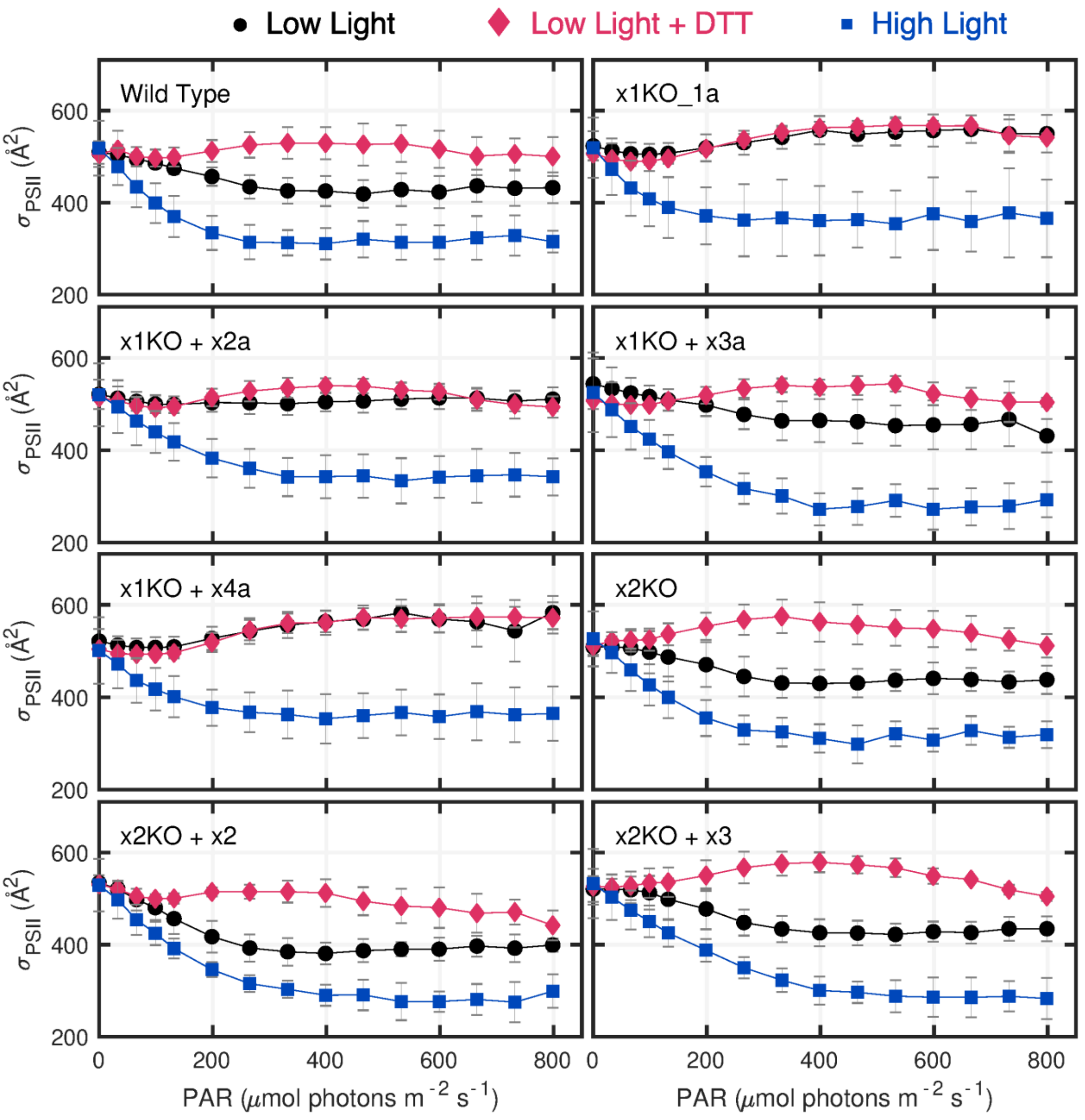

Supplementary Figure 12: Changes in σPSII during rapid light curves of Wt and mutants cultivated under low light and 24 h of high light (~ 400 µmol photons m^{-2} s^{-1})

Low light grown samples were additionally incubated with DTT. Values are the mean of six (low light cultures), five (high light cultures) or three (low light + DTT) biological replicates. SD is given. Source data are provided as a Source Data file.

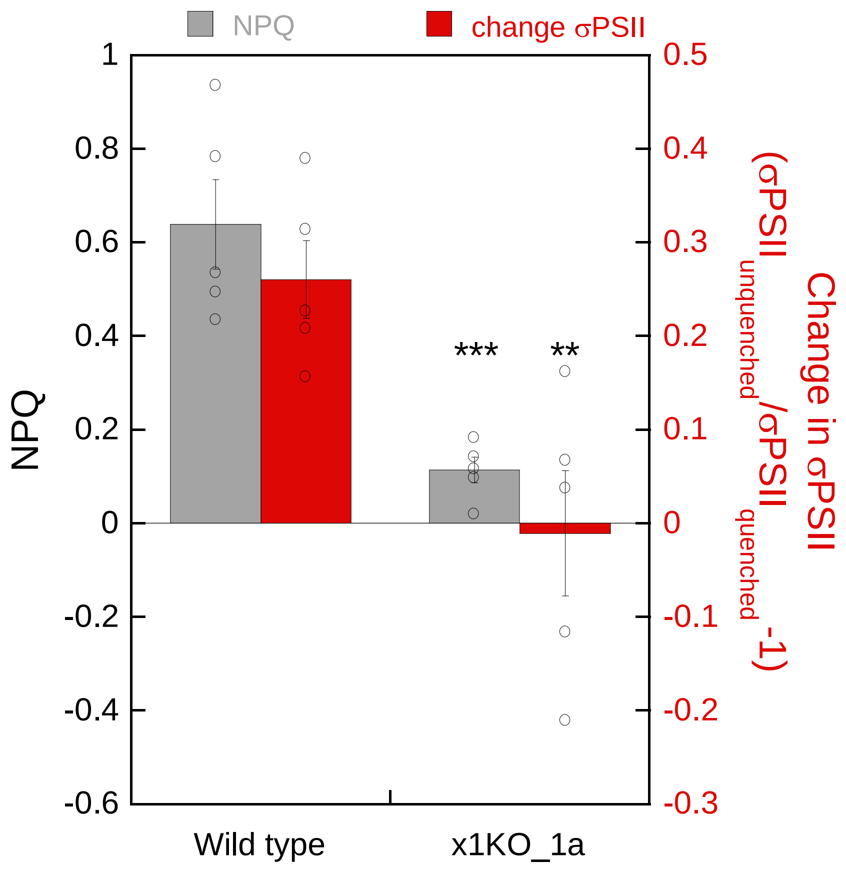

Supplementary Figure 13: Changes in NPQ and σPSII of wild type and x1KO cells upon three minutes of supra-optimal light exposure

The calculation of the change in σPSII here followed the approach of Tian et al. (2019)[1]. In order to induce qE, cells were exposed for 3 min to 1700 µmol photons m^{-2} s^{-1} (blue+red light), after which light was switched off and DCMU was added 5 s later, followed by exposure to a weak red light flash. To record σPSII of unquenched cells, DCMU was added to dark acclimated cells, after which they were also exposed to a weak red light flash. The resulting fluorescence rises were normalized according to[1], the reciprocal of the areas above the normalized fluorescence rise curve were determined as the relative functional absorption cross sections, and the changes in functional absorption cross sections were determined - in analogy to the Stern Volmer equation of NPQ - as $\sigma PSII_{unquenched}/\sigma PSII_{quenched} - 1$. More methodological details can be found in Supplementary Figure 14. Values are the mean of five independent biological replicates and SE is given. Statistical significance between Wt and x1KO cells was tested using a two-tailed unpaired Student's t-test with 8 degrees of freedom. ** $p<0.01$; *** $p<0.001$. Source data are provided as a Source Data file.

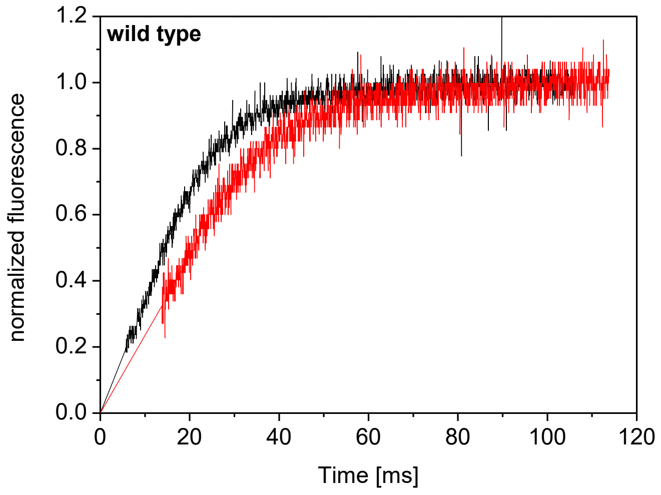

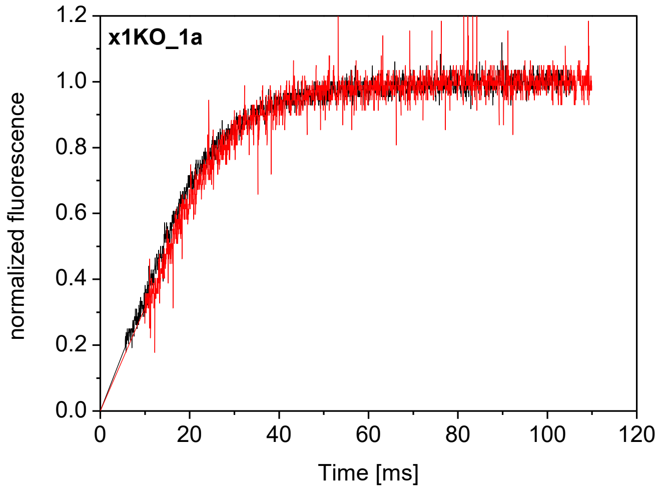

Supplementary Figure 14: Exemplary fluorescence induction traces of DCMU poisoned wild type and x1KO cells before and after supra-optimal light exposure

Cells were concentrated to 10 mg L^{-1} chlorophyll *a* and 10 µM DCMU was added in the dark, in order to record the fluorescence induction curve of unquenched cells (black trace). In order to induce qE, cells were exposed to 1700 µmol photons m^{-2} s^{-1} (blue+red light) for 3 min, then shifted to darkness for 5 s after which DCMU was added (red trace). This time span was sufficient to allow a substantial re-oxidation of Q_A^- without a pronounced relaxation of qE. Fluorescence induction curves were recorded in the fast acquisition mode with a Dual-PAM (Walz, Germany) by applying a 300 ms red light flash with an intensity of 41 µmol photons m^{-2} s^{-1}, 15 s after DCMU application. Fm was reached roughly 60 – 100 ms after flash onset. Note that DCMU treatment strongly affects the Fo values and therefore has to be corrected, as described in Tian et al. (2019)[1]. For the unquenched cells, Fo values were directly determined before treatment with DCMU, while for the quenched cells Fo' was calculated as Fo' = Fo/[1+(Fo*NPQ/Fm)][1]. Fm and Fm' were obtained from averaging the fluorescence values recorded during the 90-100 ms time span of the red light flash after DCMU application. The first 500 measured points (first 15 ms) of each fluorescence induction trace were linearly fitted and extrapolated to the corresponding Fo/Fo' value using Origin (the straight line of the fluorescence trace). The fluorescence values were then normalized to values between 0 (Fo and Fo') and 1 (Fm and Fm') and to the positive time range. The area above the fluorescence induction curve is inversely proportional to the functional absorption cross section of PSII, thus the reciprocal of this area is taken as the functional absorption cross section. Consequently, the functional absorption cross section in Wt is lower after 3 min exposure to supra-optimal light, while it is unchanged in the x1KO strain.

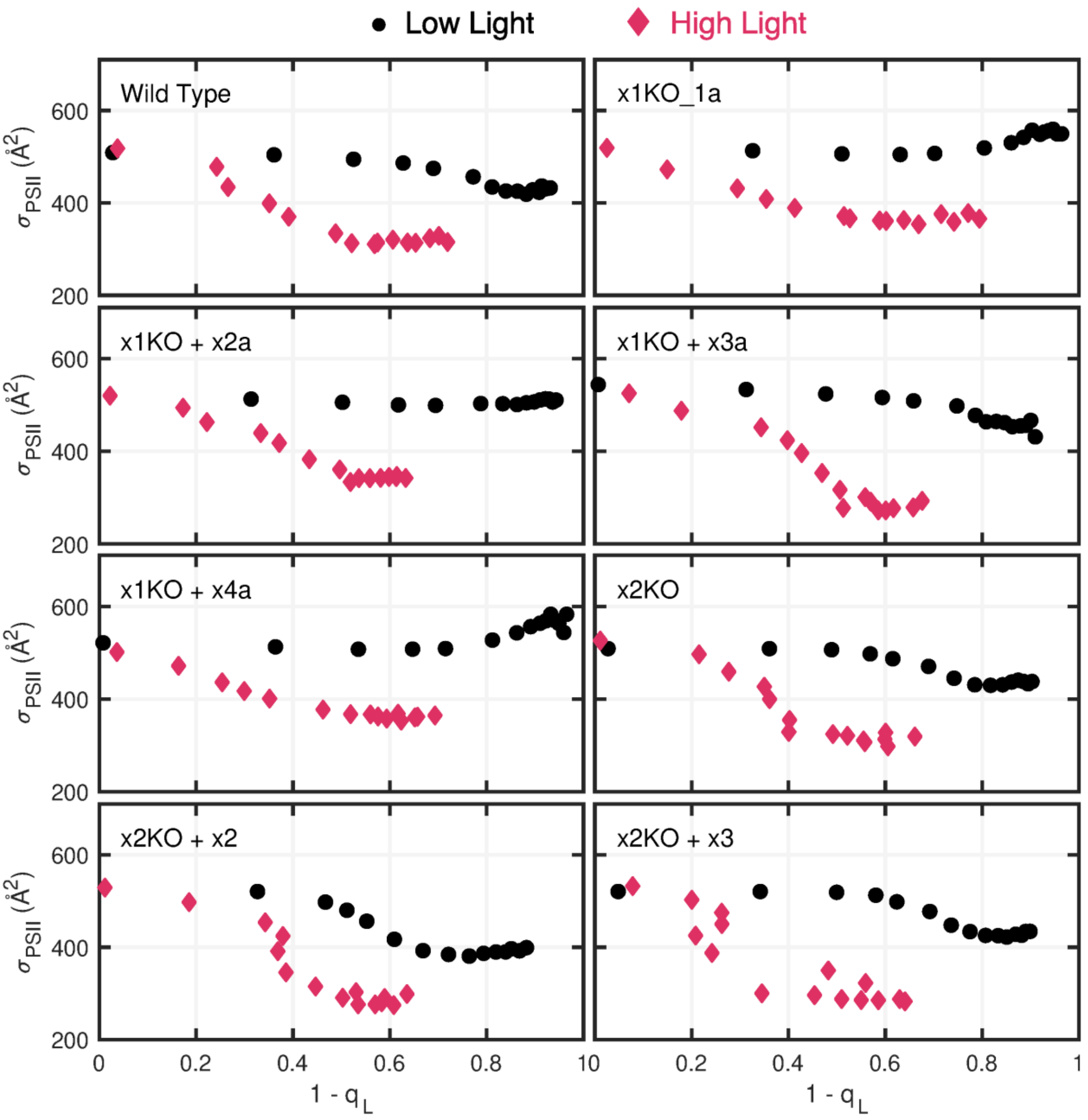

Supplementary Figure 15: Changes in σPSII *vs*. 1-qL during rapid light curves in Wt and mutants grown under low light and 24 h of high light (~ 400 µmol photons m^{-2} s^{-1})

1-qL is a proxy for the reduction state of the plastoquinone pool and thus a proxy for excitation pressure on PSII. Values are the mean of three (low light cultures) or five (high light cultures) biological replicates. Negative values were omitted. 1-qL was calculated as 1-[(Fm' - F')/(Fm'- Fo') * (Fo'/F')]. Source data are provided as a Source Data file.

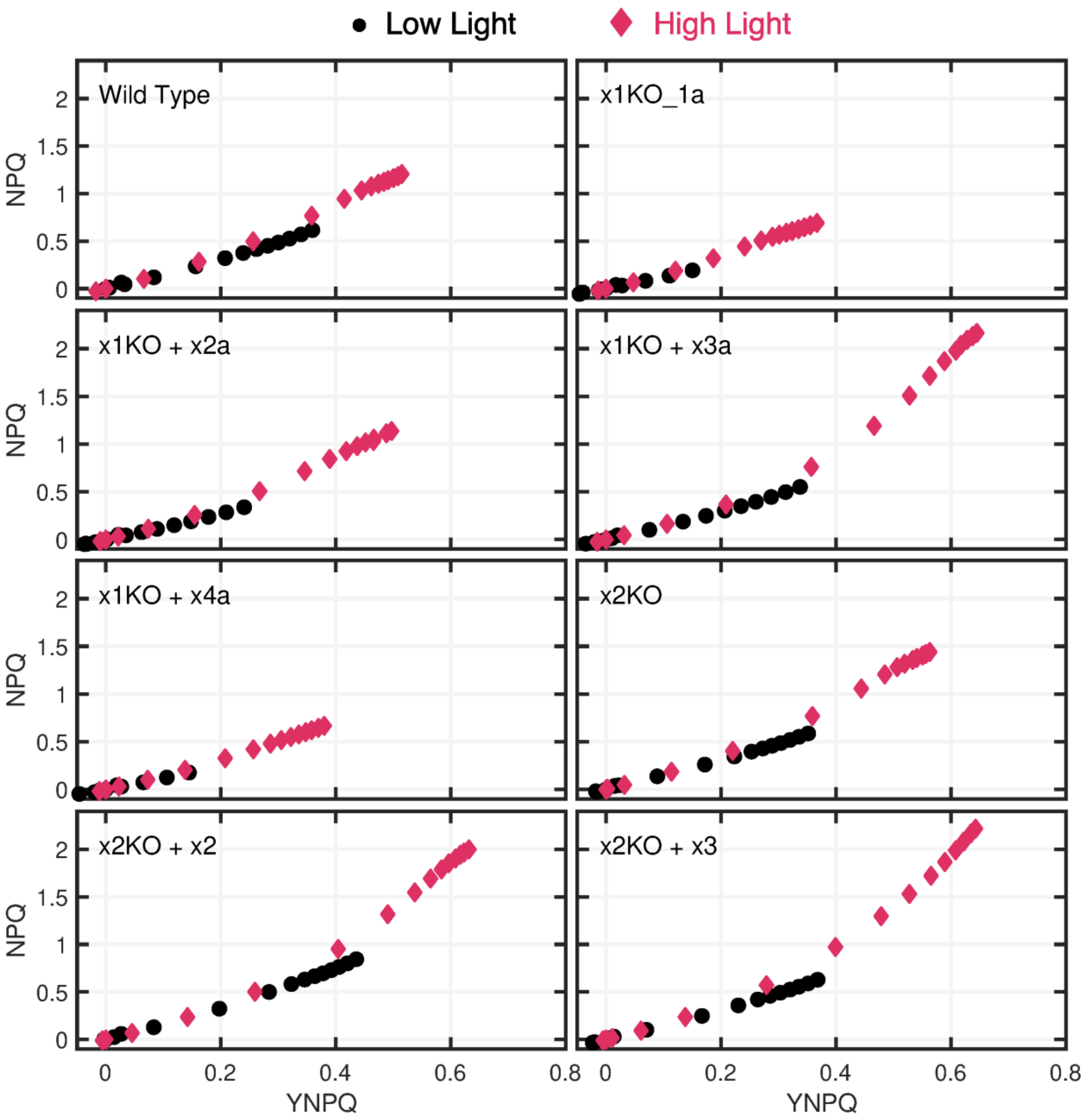

Supplementary Figure 16: NPQ *vs.* Y(NPQ) during rapid light curves in Wt and mutants cultivated under low light and 24 h of high light (~ 400 µmol photons m^{-2} s^{-1}).

Plotted points represent the mean of six (low light cultures) or five (high light cultures) biological replicates. NPQ is calculated as Fm/Fm' - 1, and Y(NPQ) is calculated as F/Fm' - F/Fm. Source data are provided as a Source Data file.

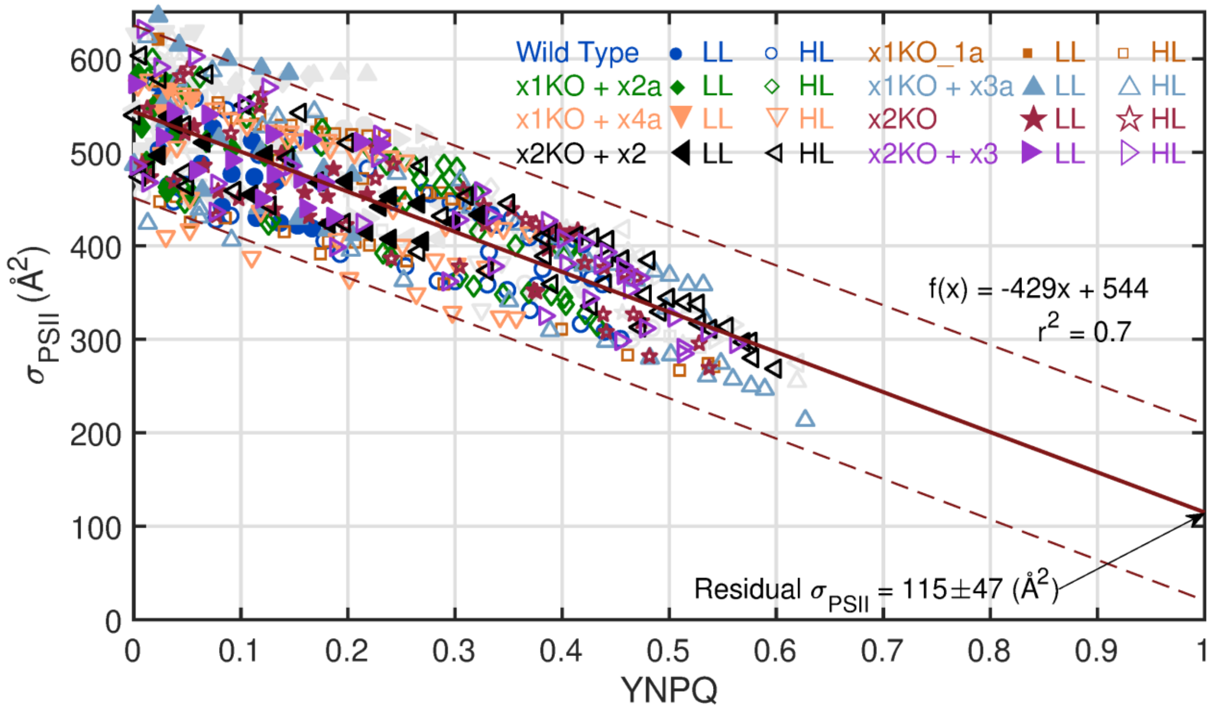

Supplementary Figure 17: σPSII$_{1s}$ *vs.* Y(NPQ)$_{1s}$ from a second measurement at each light step, following 1 second of darkness, and the corresponding linear regression

Individual data points of all measured strains cultivated both under low light (three biological replicates each), indicated by closed symbols, and high light (five biological replicates each), indicated by open symbols. Data points where an increase of Y(NPQ)$_{1s}$ did not lead to a further down-regulation of σPSII$_{1s}$ are not included in the regression calculation, but indicated in light grey. This was determined by calculating the percent change of each σPSII$_{1s}$ from its previous light step. If σPSII$_{1s}$ decreased by less than 5% of the total measured decrease for that curve while Y(NPQ)$_{1s}$ increased, it was omitted. Data points above 600 µmol photons m^{-2} s^{-1} and data points with a negative Y(NPQ)$_{1s}$ were also removed. A linear regression line, the 95% confidence interval, the regression equation and the r^2 are indicated. Source data are provided as a Source Data file.

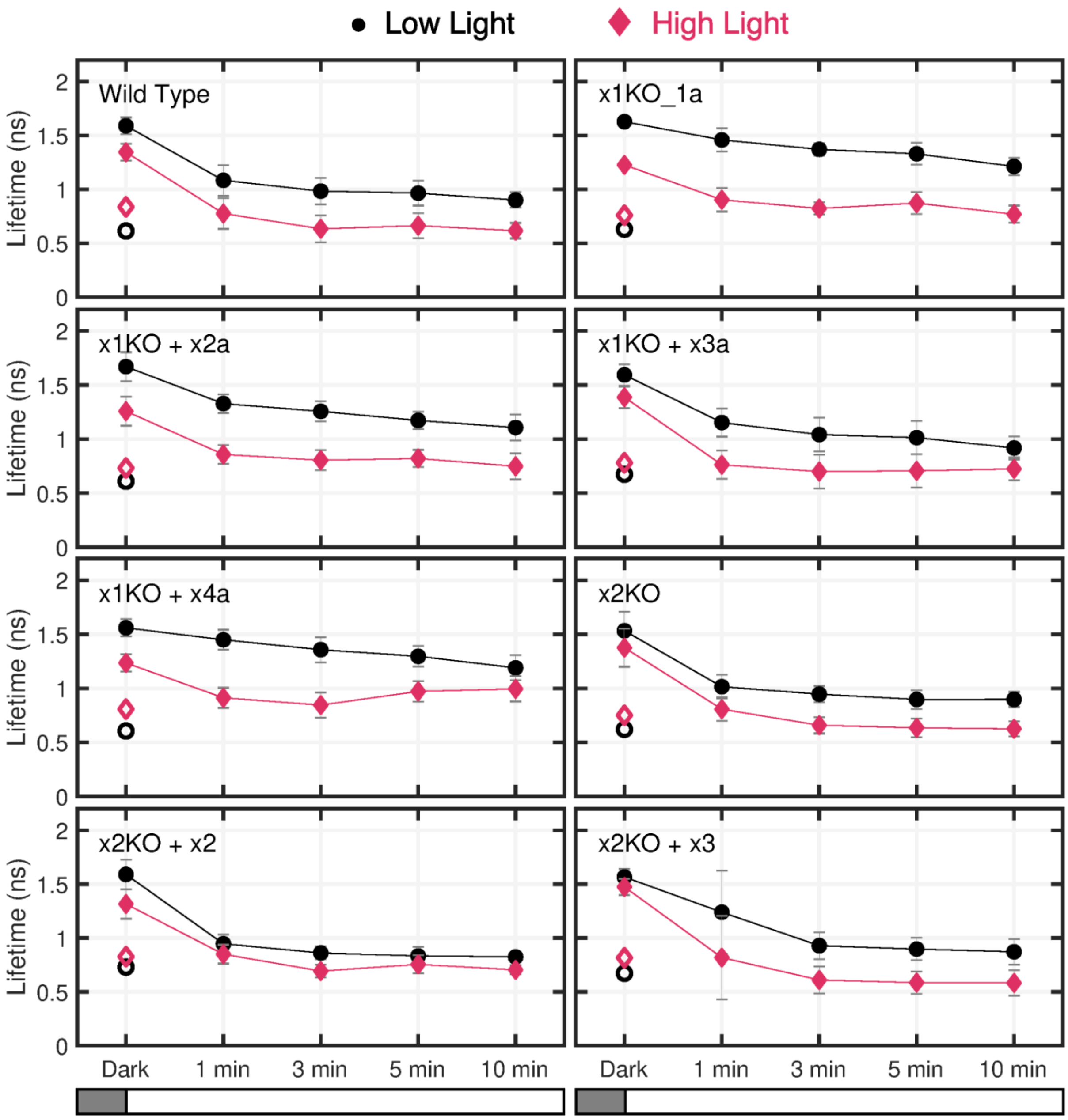

Supplementary Figure 18: Average fluorescence lifetimes during 10 min of supra-optimal light exposure in low and high light cultivated Wt and mutant strains

Open symbols in the dark represent measurements under Fo conditions. Subsequently, filled symbols represent data collected under Fm conditions. Plotted points represent the mean of six (low light cultures) or five (high light cultures) biological replicates. SD is given. Source data are provided as a Source Data file.

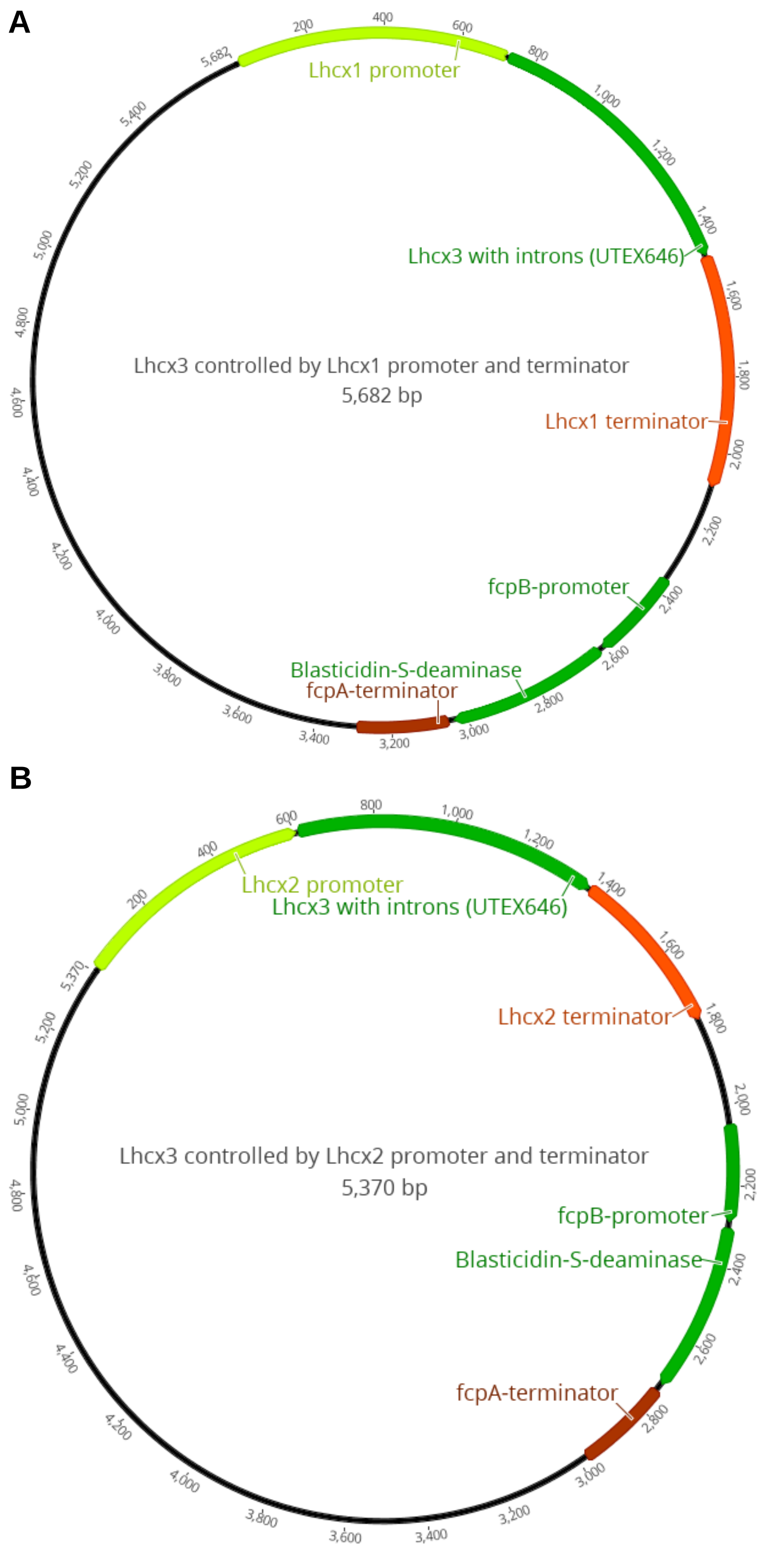

Supplementary Figure 19: Exemplary transformation vectors for the *P. tricornutum* x1KO_1a strain (A) and the x2KO strain with *Lhcx3* (B)

The vectors contain a Blasticidin-S resistance cassette. In A) the *Lhcx3* gene is under control of the *Lhcx1* promoter and terminator, while in B) it is controlled via the *Lhcx2* promoter and terminator.

GTTCTTCGT**C/A**AACACAC**T/C**GCAAA**G/A**AGAT**A/T**TACGTCCAATTCCA**A/G**CCCACTACGTACA
_CC_ATGAAATTATCCTTGGCTATCCTTGCGCTTTGCGCCAGCACTAGTGCCGCTTTCGCTCC
TTCTGTTTCCCAGAGGACGTCTGTCTCTCTCCGAGAATCATTGGACCCCACGGA**C/A**TCCA
TGTCGGAAGTGGAAGGCGCCGTGAAAGACGCGGCTCCCAAAGTCTCCGACCCTTTCGACA
GCCCTCGTGATCTTGCCGGAGTCGTCGCTCCTACCGGCTTTTTCGATCCGGCAGGCTTCGC
TGCCCGAGCCGATGCCGG**A/T**ACCATGAAGCGTTACCGGGAAGCGGAAGTTACTCACGGA
CGTGTGGGCATGATGGCCGTTGTCGGCTTTCTTGCGGGCGAAGCCGTCGAGGGATCGTC**C/**
GTTTCTCTTTGACTCGCAAGTCAGCGGACCCGCCATTACTCACCTCAACCAGATTCCTTCC
ATCTTTTGGATTCTCCTCACGGTGGGCATTGGTGCTTCCGAAGTCACGCG**C/G**GCTCAAAT
TGGTTGGGTACGTATCGGGAGTTGTTGGT**G/T**CCAACAGTTTCGCTTTGTCCGTCTCTCAC
GCGTTCTTTTCCTTTCCATTCGTTTTCCAGGTTGAACCCGAGAACGTCCCACCGGGCAAGC
CGGGTCTCCTCCGCGACGATTACGTCCCGGGTGACATTGGCTTTGATCCTCTCGGCTTGAA
GCCTTCCGACGCGCAGGCTCTCAAATCGATCCAGACCAA**A/G**GAACTGCAGAGTAAGTCA
TTTGCTGTTG**T/C**TGCTGTTACTGTCGTCC**T/A**TGGTACTATCGTGTTTACAGTTAGTTCACT
GCGTCAGAGTGTACCGTGTCATACTGTTAATCCCACCAAACTAACCGATTGAATATAACT
CGTTTTCTTTGGCTAGACGGACGTCTCGCCATGTTGGCGGCGGCTGGGTGCATGGCTCAG
GAATTGGCCAACGGAAAGGGCATTCTCGAAAACCTTGG**T/G**CTCTAA_GGAATAT**A/G**ACGTT_
CCAGTGTTTTGAACACTAGGCGCGATGGACAACAGAACGATTAGGCTAGCAACGAAAAGAAGCA
AAATACAGTAGAAATCAAGTAATTCTCCCACTTTGT**T/G**CAAA.

Supplementary Figure 20: *Lhcx2* gene sequence of *P. tricornutum* strain 4

Bases before and after “/”, underlined and in bold, indicate alternating bases in the two alleles (in total 16, the first base always referring to allele 1), and the untranslated region is indicated in italics. Green letters indicate introns, while blue letters indicate the TALEN binding site.

```
WT        ATGAAATTATCCTTGGCTATCCTTGCGCTTTGCGCCAGCACTAGTGCCGCTTTCGCTCCT    60
x2KO      ATGAAATTATCCTTGGCTATCCTTGCGCTTTGCGCCAGCACTAGTGCCGCTTTCGCTCCT    60
          ************************************************************
WT        TCTGTTTCCCAGAGGACGTCTGTCTCTCTCCGAGAATCATTGGACCCCACGGAATCCATG    120
x2KO      TCTGTTTCCCAGAGGACGTCTGTCTCTCTCCGAGAATCATTGGACCCCACGGAATCCATG    120
          ************************************************************
WT        TCGGAAGTGGAAGGCGCCGTGAAAGACGCGGCTCCCAAAGTCTCCGACCCTTTCGACAGC    180
x2KO      TCGGAAGTGGAAGGCGCCGTGAAAGACGCGGCTCCCAAAGTCTCCGACCCTTTCGACAGC    180
          ************************************************************
WT        CCTCGTGATCTTGCCGGAGTCGTCGCTCCTACCGGCTTTTTCGATCCGGCAGGCTTCGCT    240
x2KO      CCTCGTGATCTTGCCGGAGTCGTCGCTCCTACCGGCTTTTTCGATCCGGCAGGCTTCGCT    240
          ************************************************************
WT        GCCCGAGCCGATGCCGGTACCATGAAGCGTTACCGGGAAGCGGAAGTTACTCACGGACGT    300
x2KO      GCCCGA------------------------------------------------------    246
          ******
WT        GTGGGCATGATGGCCGTTGTCGGCTTTCTTGCGGGCGAAGCCGTCGAGGGATCGTCGTTT    360
x2KO      ------------------------------------------------------------    246

WT        CTCTTTGACTCGCAAGTCAGCGGACCCGCCATTACTCACCTCAACCAGATTCCTTCCATC    420
x2KO      ------------------------------------------------------------    246

WT        TTTTGGATTCTCCTCACGGTGGGCATTGGTGCTTCCGAAGTCACGCGGGCTCAAATTGGT    480
x2KO      ------------------------------------------------------------    246

WT        TGGGTACGTATCGGGAGTTGTTGGTTCCAACAGTTTCGCTTTGTCCGTCTCTCACGCGTT    540
x2KO      ------------------------------------------------------------    246

WT        CTTTTCCTTTCCATTCGTTTTCCAGGTTGAACCCGAGAACGTCCCACCGGGCAAGCCGGG    600
x2KO      ------------------------------------------------------------    246

WT        TCTCCTCCGCGACGATTACGTCCCGGGTGACATTGGCTTTGATCCTCTCGGCTTGAAGCC    660
x2KO      ------------------------------------------------------------    246

WT        TTCCGACGCGCAGGCTCTCAAATCGATCCAGACCAAGGAACTGCAGAGTAAGTCATTTGC    720
x2KO      ------------------------------------------------------------    246

WT      TGTTGCTGCTGTTACTGTCGTCCATGGTACTATCGTGTTTACAGTTAGTTCACTGCGTCA      780
x2KO      ------------------------------------------------------------    246

WT        GAGTGTACCGTGTCATACTGTTAATCCCACCAAACTAACCGATTGAATATAACTCGTTTT    840
x2KO      ------------------------------------------------------------    246

WT        CTTTGGCTAGACGGACGTCTCGCCATGTTGGCGGCGGCTGGGTGCATGGCTCAGGAATTG    900
x2KO      ------------------------------------------------------------    246

WT        GCCAACGGAAAGGGCATTCTCGAAAACCTTGGGCTCTAAGGAATATGACGTTCCAGTGTT    960
x2KO      ------------------------------------------------------------    246

WT        TTGAACACTAGGCGCGATGGACAACAGAACGATTAG    996
x2KO      ------------------------------------    246
```

Supplementary Figure 21: Alignment of *Lhcx2* allele 2 amplified from wild type and the x2KO strain In the x2KO strain, the *Lhcx2* gene is deleted from base pair 247 up to the end of the gene. The same deletion can also be found in the x2KO+x2 line. The binding sites for the forward and reverse TALEN construct used to induce the knockout in the *Lhcx2* gene are indicated in blue and bold.

Supplementary Table 1: TALENs used to target *Lhcx1* and *Lhcx2*

Gene	RVD forward TALEN	Backbone vector forward TALEN	RVD reverse TALEN	Backbone vector reverse TALEN	+ strand sequence (in minuscule: spacer sequence)
Lhcx1	HD NN HD NG NN HD HD NI HD HD NI NG HD HD NG NG NN HD NG	pM9_fcpA_NG (#90420)	NG NN NG NG NG NN NN NN HD HD NN NN NI NN HD NN NI NI NI	pM9_NR_NI (#90422)	CGCTGCCACCATCCTTGCT cttatcggctctgccgctgc TTTCGCTCCGGCCCAAACA
Lhcx1	NI HD NI HD NN NI HD HD NN NG NN NN NG HD HD NG NG HD NG	pM9_fcpA_NG (#90420)	NN NN HD NG HD HD NI NI HD NG NI NN NI NG HD NG NG HD NG	pM9_NR_NG (#90423)	ACACGACCGTGGTCCTTCT tacataaacctgcagaa AGAAGATCTAGTTGGAGCCA
Lhcx2	HD NH NI NG HD HD NH NH HD NI NH NH HD NG NG HD NH HD NG	pM9_fcpA_NG (#90420)	HD HD HD NH NH NG NI NI HD NH HD NG NG HD NI NG NH NH NG	pM9_NR_NG (#90423)	CGATCCGGCAGGCTTCGCT gcccgagccgatgccggg ACCATGAAGCGTTACCGGG

Indicated are the repeat variable di-residues (single letter amino acid code) characteristic for the respective TALE monomers, the used backbone vectors (available at Addgene with the indicated vector code) and the targeted DNA sequence.

Supplementary Table 2: Cloned DNA sequences in order to complement the x1KO and x2KO lines

Cloned DNA sequence	Bases
Lhcx1 promotor, used for *Lhcx2*, *Lhcx3* and *Lhcx4*	CCTTAACTGGGTCAGTACAGCGTGTTGATTGTGAACAAAACCAATGGCCAGACGGATTCGCAAGTTCACGACAG<br>CTTTCGACGGTGAGAATGATGGTATCGCTTCGGGTCGGGTCGGTACTTTGCGGGACTGGCAACGTCGGCAGAGG<br>GACTCACGGTCACTTCACTGTCTGTATCGACTGACGACTGACTCGTCTGATTCACTGTCAGGTTTCTGATCTAAGT<br>TTATCAAATGCTACTACTTGAGGTAGTCGTATCATTTGAAACTGCAACTTACAAAATCGAAGGTCCTTGCAACAT<br>TTCTGACATGGGTAATTTCATAGGATGTTACAATGTCGAGCTTGTATCACTCGGATACAACAGACCACTTTAGGA<br>AAGGGATTTTTTTTGTCGAAAATAGGATGACATCAACAGGAAGTACGTTCATAGCCCTTTATTTATAATTGGAT<br>GGGTCAATCCGACGTACGCATGTGTCTTTTGACCTTGTCATCCATAACCAGATTGACCCGTCACTGTGCAATACG<br>TCACGCCAATGAAAGACCCCCGGATAAAGGGCCTAAAATTCAGTCTCGTGCAAAACACGCAGGATGATGCACCT<br>GAACAGGTGACGTGTTCGCGCAGTCGAATGAGTTCCGAAGCCATCAGAGGCGAATTTATTTTGCAACTGTTTGA<br>CCCCATAAAACCATTCCTAGGAGATTTTGATAAACACTTGAAAATAACG
Lhcx1 terminator	AGAGTGCATCCATCCAAAACTGGAGGTATGGCTAGTCACATGCACAGAGTTAACAACCAATCCCTTTTCAAGCG<br>AAGCTCGCATGGTCTGCCGCAACAAAATTTAGTTGATGCCACCACATAGAACGCTGTAACATAGCCTAAAGCGC<br>AGGTTTTATAATCAAAATGTATGGAAGCAACTTTGTCCTTTTGAAGCGGCTTTCTCAACGTTTGGAGATAGACAA<br>TATCTTGGAGATTTGTGCGTCTGGGTTTGATTGGTTTGCAATCCTTGTATAGCATTTACCAATTGTGCCGAAGTCG<br>ACCGAACTGTTGTTTCCGATTATACCCATGGGTGCGATACACAAAGAGAAATCCATGGAACAAGACTTTCGAAA<br>ATTCGTGGTAGTTCCGAAGGAACGCACTGACTGTGACTTGTGACTGACTGTCATAGCTAGCACGCACTGACTGTG<br>ACTTGTGACTGACTGTCATAACTAGCTAAAACAGGCATCACAGTCAGGCGGTTGTACTGATAGGGCAGGCAAAG<br>TACAGGCACCAAAATTCGGACGAAACAACGCGACAAGATCAGTACCGCGTACGGCTTTTTGTTTACGGAACTAT<br>TCCCACCTTATCTGGACCATG
Lhcx1 promoter for modified *Lhcx1* gene	CCTTAACTGGGTCAGTACAGCGTGTTGATTGTGAACAAAACCAATGGCCAGACGGATTCGCAAGTTCACGACAG<br>CTTTCGACGGTGAGAATGATGGTATCGCTTCGGGTCGGGTCGGTACTTTGCGGGACTGGCAACGTCGGCAGAGG<br>GACTCACGGTCACTTCACTGTCTGTATCGACTGACGACTGACTCGTCTGATTCACTGTCAGGTTTCTGATCTAAGT<br>TTATCAAATGCTACTACTTGAGGTAGTCGTATCATTTGAAACTGCAACTTACAAAATCGAAGGTCCTTGCAACAT<br>TTCTGACATGGGTAATTTCATAGGATGTTACAATGTCGAGCTTGTATCACTCGGATACAACAGACCACTTTAGGA<br>AAGGGATTTTTTTTGTCGAAAATAGGATGACATCAACAGGAAGTACGTTCATAGCCCTTTATTTATAATTGGAT<br>GGGTCAATCCGACGTACGCATGTGTCTTTTGACCTTGTCATCCATAACCAGATTGACCCGTCACTGTGCAATACG<br>TCACGCCAATGAAAGACCCCCGGATAAAGGGCCTAAAATTCAGTCTCGTGCAAAACACGCAGGATGATGCACCT<br>GAACAGGTGACGTGTTCGCGCAGTCGAATGAGTTCCGAAGCCATCAGAGGCGAATTTATTTTGCAACTGTTTGA<br>CCCCATAAAACCATTCCTAGGAGATTTTGATAAACACTTGAAAATAACGGAATTCAGGCCT
Lhcx2 promotor	CTCACAGTAACATAGCATTGTCGAACTAACCTGTAATACTAACGTGACGTATGAATGAATCGTGTATGACGTTCC<br>GGTGTCGAGTCACTATCATACCATTGGTGATACGATATCACGTGAGATATCACTGATATCTCACGTCAATAGACA<br>AGCTGCTTTTCAATGGGTGAAAACAACCACAATACGGAACAAGTTCCTGTAACTCTAACTATTGATTGTAGTTGG<br>TGAAGAAAGCATTTTCTTGACTGTGGGTACATGGAATACATCCTTTTTTGATAAATGTACAAACAAAGACTCATT<br>TAGTCATAGTCAAGCGCAGTGCGTACTTGCCTGTGAAAAAATCGAGCTCTGAAAGGGTTAAAGTTCAAGTTGTA<br>CAAGGTAGAGCTGGTTTGCGTGTGTCAGGGCGTGGCCTACCTGACTCATGCATTCCGCGTGCCACAAAAACTCA<br>CAGTCAGAGAGCCACTCCGAGAATCCTCCAGAATTTCGTGGAAGATTTTTTCGTCATCTTTTTCCAGTTCTCCCCT<br>TAGCTCTCATAGTTTCGTTTGCGTTCTTCGTAAACACACTGCAAAGAGATTTACGTCCAATTCCAACCCACTACGT<br>ACACC
Lhcx2 terminator	GGAATATAACGTTCCAGTGTTTTGAACACTAGGCGCGATGGACAACAGAACGATTAGGCTAGCAACGAAAAGA<br>AGCAAAATACAGTAGAAATCAAGTAATTCTCCCACTTTGTTCAAACACGCTGACAATTCTTTATGTTAGTGTAGC<br>CCTTTTAATCGTGCACGTGTTCCGAGTCATTGCCCGGGACGTTTGTCACAGCACATGAAACGAGAAACTTCACCA<br>GACAGTTGGCTCACAATCAATTTCATGAGTTTCTACATTGATTGACTACCCAAGCTCCGTCCATCGTTGCAGATTT<br>CATGCGTATGGAATCATTGAATCAAGTATCGTGAAGTTTCCAACGAGGAACTTGCGGTCACACTATTCTTCGTAC<br>CAACCACGGAAGCTCCTGGTCCATCATGGCAGATCTCACGCTGGTAG
Lhcx1 gene modified without introns	ATGAAGTTTGCCGCAACTATATTGGCCCTTATCGGCTCTGCCGCTGCGTTTGCGCCTGCACAGACGAGCCGTGCG<br>TCTACTAGCCTTCAGTACGCGAAGGAGGACTTGGTGGGGGCTATTCCTCCGGTCGGATTCTTCGACCCTCTTGGA<br>TTCGCTGACAAGGCCGATTCCCCCACTTTGAAGCGATACCGTGAAGCTGAGCTCACCCACGGACGTGTTGCCATG<br>CTTGCCGTCGTTGGATTCCTTGTCGGCGAGGCGGTAGAAGGTTCGTCGTTCCTCTTCGATGCTTCTATCTCTGGCC<br>CGGCCATCACCCACCTTTCTCAAGTCCCGGCCCCCTTCTGGGTCCTCCTCACTATTGCTATCGGTGCTTCCGAACA<br>GACCCGTGCCGTGATCGGCTGGGTGGATCCCGCCGATGCCCCGGTTGACAAGCCCGGTCTTCTCCGTGACGACTA<br>CGTCCCGGGTGACCTCGGATTCGACCCTCTCGGCCTCAAGCCTTCTGACCCGGAAGAACTGATCACTCTCCAGAC<br>GAAGGAACTTCAGAACGGACGTCTTGCTATGCTTGCCGCTGCCGGTTTCATGGCTCAGGAGCTTGTCAACGGGA<br>AGGGAATCCTTGAGAATCTTCAGGGTTAA

Lhcx2 gene	ATGAAATTATCCTTGGCTATCCTTGCGCTTTGCGCCAGCACTAGTGCCGCTTTCGCTCCTTCTGTTTCCCAGAGGA<br>CGTCTGTCTCTCTCCGAGAATCATTGGACCCCACGGAATCCATGTCGGAAGTGGAAGGCGCCGTGAAAGACGCG<br>GCTCCCAAAGTCTCCGACCCTTTCGACAGCCCTCGTGATCTTGCCGGAGTCGTCGCTCCTACCGGCTTTTTCGATC<br>CGGCAGGCTTCGCTGCCCGAGCCGATGCCGGTACCATGAAGCGTTACCGGGAAGCGGAAGTTACTCACGGACGT<br>GTGGGCATGATGGCCGTTGTCGGCTTTCTTGCGGGCGAAGCCGTCGAGGGATCGTCGTTTCTCTTTGACTCGCAA<br>GTCAGCGGACCCGCCATTACTCACCTCAACCAGATTCCTTCCATCTTTTGGATTCTCCTCACGGTGGGCATTGGTG<br>CTTCCGAAGTCACGCGGGCTCAAATTGGTTGGGTACGTATCGGGAGTTGTTGGTTCCAACAGTTTCGCTTTGTCC<br>GTCTCTCACGCGTTCTTTTCCTTTCCATTCGTTTTCCAGGTTGAACCCGAGAACGTCCCACCGGGCAAGCCGGGTC<br>TCCTCCGCGACGATTACGTCCCGGGTGACATTGGCTTTGATCCTCTCGGCTTGAAGCCTTCCGACGCGCAGGCTC<br>TCAAATCGATCCAGACCAAGGAACTGCAGAGTAAGTCATTTGCTGTTGCTGCTGTTACTGTCGTCCATGGTACTA<br>TCGTGTTTACAGTTAGTTCACTGCGTCAGAGTGTACCGTGTCATACTGTTAATCCCACCAAACTAACCGATTGAA<br>TATAACTCGTTTTCTTTGGCTAGACGGACGTCTCGCCATGTTGGCGGCGGCTGGGTGCATGGCTCAGGAATTGGC<br>CAACGGAAAGGGCATTCTCGAAAACCTTGGTCTCTAA
Lhcx2 gene modified	ATGAAATTATCCTTGGCTATCCTTGCGCTTTGCGCCAGCACGTCCGCAGCATTTGCACCAAGCGTCAGTCAACGA<br>ACAAGCGTTAGCTTGAGGGAGAGCCTTGATCCAACAGAGAGTATGTCGGAAGTGGAAGGCGCCGTGAAAGACG<br>CGGCTCCCAAAGTCTCCGACCCTTTCGACAGCCCTCGTGATCTTGCCGGAGTCGTCGCTCCTACCGGCTTTTTTGA<br>CCCAGCTGGATTTGCAGCCCGAGCCGATGCCGGAACAATGAAAAGATATAGAGAAGCGGAAGTTACTCACGGA<br>CGTGTGGGCATGATGGCCGTTGTCGGCTTTCTTGCGGGCGAAGCCGTCGAGGGATCGTCCTTTCTCTTTGACTCG<br>CAAGTCAGCGGACCCGCCATTACTCACCTCAACCAGATTCCTTCCATCTTCTGGATCTTGTTGACAGTGGGCATT<br>GGTGCTTCTGAGGTTACACGTGCACAGATTGGTTGGGTACGTATCGGGAGTTGTTGGTGCCAACAGTTTCGCTTT<br>GTCCGTCTCTCACGCGTTCTTTTCCTTTCCATTCGTTTTCCAGGTTGAACCCGAGAACGTCCCACCGGGCAAGCCG<br>GGTCTCCTCCGCGACGATTACGTCCCGGGTGACATTGGCTTTGATCCTCTCGGCTTGAAGCCTTCCGACGCGCAG<br>GCTCTCAAATCGATCCAGACCAAAGAACTGCAGAGTAAGTCATTTGCTGTTGTTGCTGTTACTGTCGTCCTTGGT<br>ACTATCGTGTTTACAGTTAGTTCACTGCGTCAGAGTGTACCGTGTCATACTGTTAATCCCACCAAACTAACCGAT<br>TGAATATAACTCGTTTTCTTTGGCTAGACGGACGTCTCGCCATGTTGGCGGCGGCTGGGTGCATGGCTCAGGAAT<br>TGGCCAACGGAAAGGGCATTCTCGAAAACCTTGGTCTCTAA
Lhcx3 gene	ATGAAGCGCATCGCCGCTATCGCCCTTCTCGCCACTACGGCGTCCGCCTTTAACGCATTCGGTGCCGCCAAGAAG<br>GCTGCGCCCAAAAAGCCGGTACGTCTACGCGAACCTTGGCATTTTCCAAGTACCACCGTGTCGGTGTGTTACAGC<br>TAGTGTTGTCCGCGATTGCATTCCTCACGAACCACACTCGTTCCGCTTTGTCCCGGCAGGTATTCTCGATCGAAA<br>CGATCCCCGGTGCGCTCGCTCCCGTTGGTATCTTTGATCCCCTCGGTTTCGCCGCCAAGGCCGACGAGTCCACCC<br>TGAAGCGATACCGCGAAGCCGAGCTCACCCACGGACGGGTGGCCATGCTCGCTACCGTTGGCTTCTTGGTCGGT<br>GAAGCCGTGGAAGGATCTTCCTTCCTCTTTGATGCTTCTATCAAAGGACCTGCTATTTCTCATCTCGCCCAAGTGC<br>CGACTCCGTTCTGGGTTCTCTTGACCATTTTCATCGGGGCCGCGGAACAGACCCGTGCCGTCATCGGCTGGCGGG<br>ATCCTTCCGACGTACCCTTCGACAAGCCCGGTCTCTTGAACGAGGACTACACCCCGGGTGACATTGGCTTTGATC<br>CTCTCGGACTCAAGCCAACGGATGCGGAAGAACTCAGGGTTCTACAGACCAAGGAACTCCAGAACGGACGCTTG<br>GCCATGCTTGCTGCCGCTGGATTCATGGCGCAGGAACTCGTGGACGGCAAGGGAATCCTGGAACACCTCCTCTA<br>A
Lhcx4 gene	ATGAAATTGTTCACGATCTTCTTGCCCCTGGTTTTAGTGGGAACCGCCGCTGGCTTTGCCTCTGGCCCGTTCTCTA<br>AGAAGACTTCTCCGTCACCAGTAAGTTGTCTCACACCGAGACCAATCTCATGTCCACTTGACGAAAGCATTGTCT<br>TATGATCATAATTTCTCGTTTACTCTGCAGGAGGTATCAATTGAAAGTATGCCTGGTATCGTGGCGCCCACTGGA<br>TTCTTTGATCCACTCCGCTTCGCTGAAAGGGCCCCGTCGAACACACTTAAGCGCTACCGCGAGTGTGAACTAACG<br>CATGGCCGCGTTGCTATGTTGGCAACGGTGGGTTTCCTCGCCGGCGAAGCGGTTCAAAATACGAACTTTCTATGG<br>AACGCCCAAGTTTCGGGGCCTGCTATAACGCATATTCCACAGATTCCAGCAACTTTTTGGGTCTTGCTCACCCTG<br>TTTATCGGTGTGGCCGAATTGTCACGTGCGCAAACTGCCATGGTTCCCCCCAGTGACATTCCCGTGGGTAAGGCT<br>GGCCGAATGCGTGAAGATTACAATCCTGGGGACATTGGATTCGACCCTCTCAATTTAATGCCCGAAAGTTCCGA<br>GGAGTTCTATAGGTTGCAGACTAAAGAACTACAGAATGGGCGTTTGGCCATGCTGGGTGCTGCAGGTTTCTTGG<br>CTCAAGAAGCAGTTAATGGGAAGGGTATTTTGGAGAATTTGTTTGGCTAG

For the modified *Lhcx1* gene, 12 bases coding for the restriction enzymes recognition sites of *Eco*RI and *Stu*I had been introduced before the start of the coding sequences (i.e. at the end of the promoter) due to cloning strategy reasons.

Supplementary Table 3: Primers used in this study

Name	Forward primer sequence	Reverse primer sequence
*Lhcx1*_all-fw	5’-CTCTCCAGACGAAGGAAC-3’	
*Lhcx1*_all-rev		5’-GATTCTCAAGGATTCCC-3’
*Lhcx1*_Wt-fw	5’-CTGCCACCATCCTTGCTCTT-3’	
*Lhcx1*_Wt-rev		5’-GACGAACCTTCTACCGCCTC-3’
*Lhcx1*_comp-fw	5’-GCCGCAACTATATTGGCCCT-3’	
*Lhcx2*_allele1-fw	5’-AAGAGATATACGTCCAATTCCAA-3’	
*Lhcx2*_allele1-rev		5’-AAGGACGACAGTAACAGCAA-3’
*Lhcx2*_allele2-fw	5’-CGTAAACACACCGCAAAA-3’	
*Lhcx2*_allele2-rev		5’-AAATGACTTACTCTGCAGTTCC-3’
*Lhcx2*_prom-fw	5‘-CTCACAGTAACATAGCATTGTCG-3‘	
*Lhcx2*_term-rev		5‘-CTACCAGCGTGAGATCTGC-3‘
*FcpA*_Lhcx1-fw	5’-GGCTGCAGGACGCAATG-3’	
*FcpA*_Lhcx1-rev		5’- AGGGCCAATATAGTTGCGGC-3’
*Lhcx1*_qPCR_fw	5’-AAGGTTCGTCGTTCCTCTTCG-3’	
*Lhcx1*_qPCR_rev		5’-CGGAAGCACCGATAGCAATAGT-3’
*Lhcx2*_qPCR_fw	5’-CGCCATTACTCACCTCAACCAG-3’	
*Lhcx2*_qPCR_rev		5’-TCAACCCAACCAATTTGAGC-3’
*Lhcx3*_qPCR_fw	5’-TCTTGAACGAGGACTACACCCC-3’	
*Lhcx3*_qPCR_rev		5’-TCCGTTCTGGAGTTCCTTGGT-3’
*Lhcx4*_qPCR_fw	5’-ATGGCCGCGTTGCTATGTT-3’	
*Lhcx4*_qPCR_rev		5’-TATAGCAGGCCCCGAAACTTG-3’
*18s*_qPCR_fw	5’-TGCCCTTTGTACACACCGC-3’	
*18s*_qPCR_rev		5’-AAGTTCTCGCAACCAACACCA-3’

Primers use for qPCR analyses are indicated as “*gene name*_qPCR_fw/rev”.

Supplementary Reference:

1: Tian, L. *et al.* pH dependence, kinetics and light-harvesting regulation of nonphotochemical quenching in *Chlamydomonas*. *Proc Natl Acad Sci USA* **116**, 8320-8325, doi:https://doi.org/10.1073/pnas.1817796116 (2019).
