# Supplementary material for: Lhcx proteins provide photoprotection via thermal dissipation of absorbed light in the diatom Phaeodactylum tricornutum
Source: Nat Commun. 2019 Sep 13;10:4167. doi: 10.1038/s41467-019-12043-6 (PMC6744471; doi:10.1038/s41467-019-12043-6)
Supplement: Supplementary file 3 — Reporting Summary [file 41467_2019_12043_MOESM3_ESM.pdf]

nature research

Corresponding author(s): Dr. Bernard Lepetit

Last updated by author(s): Aug 4, 2019

Reporting Summary

Nature Research wishes to improve the reproducibility of the work that we publish. This form provides structure for consistency and transparency in reporting. For further information on Nature Research policies, see Authors & Referees and the Editorial Policy Checklist.

Statistics

For all statistical analyses, confirm that the following items are present in the figure legend, table legend, main text, or Methods section.

n/a	Confirmed	
☐	☒	The exact sample size (*n*) for each experimental group/condition, given as a discrete number and unit of measurement
☐	☒	A statement on whether measurements were taken from distinct samples or whether the same sample was measured repeatedly
☐	☒	The statistical test(s) used AND whether they are one- or two-sided<br>*Only common tests should be described solely by name; describe more complex techniques in the Methods section.*
☒	☐	A description of all covariates tested
☒	☐	A description of any assumptions or corrections, such as tests of normality and adjustment for multiple comparisons
☐	☒	A full description of the statistical parameters including central tendency (e.g. means) or other basic estimates (e.g. regression coefficient) AND variation (e.g. standard deviation) or associated estimates of uncertainty (e.g. confidence intervals)
☐	☒	For null hypothesis testing, the test statistic (e.g. *F*, *t*, *r*) with confidence intervals, effect sizes, degrees of freedom and *P* value noted<br>*Give P values as exact values whenever suitable.*
☒	☐	For Bayesian analysis, information on the choice of priors and Markov chain Monte Carlo settings
☒	☐	For hierarchical and complex designs, identification of the appropriate level for tests and full reporting of outcomes
☒	☐	Estimates of effect sizes (e.g. Cohen's *d*, Pearson's *r*), indicating how they were calculated

Our web collection on statistics for biologists contains articles on many of the points above.

Software and code

Policy information about availability of computer code

Data collection	Data collection has been performed with different machines (Fluorometers, HPLC, qPCR, Blotting device) indicated in the text. The respective data are then used to plot graphs with standard plotting software (see below).
Data analysis	While most of the data were analyzed using standard commercial software (Excel, Matlab, SigmaPlot, Kaleidagraph, Origin, CLC Genomics, Geneious R9), specific software for qPCR (PCR Miner and REST 2006) and whole genome sequencing is available freely and is indicated in the manuscript.

For manuscripts utilizing custom algorithms or software that are central to the research but not yet described in published literature, software must be made available to editors/reviewers. We strongly encourage code deposition in a community repository (e.g. GitHub). See the Nature Research guidelines for submitting code & software for further information.

Data

Policy information about availability of data

All manuscripts must include a data availability statement. This statement should provide the following information, where applicable:

- Accession codes, unique identifiers, or web links for publicly available datasets
- A list of figures that have associated raw data
- A description of any restrictions on data availability

Source data for the figures and supplementary figures are provided with the article. Any further data related to the article is available from the corresponding author upon reasonable request. Whole genome sequencing reads for Pt4, x1KO_1a, x1KO_1b and x1KO_2 are deposited at the European Nucleotide archive (ENA) under the following accession code: "PRJEB33825[https://www.ebi.ac.uk/ena/data/view/PRJEB33825]".

Field-specific reporting

Please select the one below that is the best fit for your research. If you are not sure, read the appropriate sections before making your selection.

- [x] Life sciences
- [ ] Behavioural & social sciences
- [ ] Ecological, evolutionary & environmental sciences

For a reference copy of the document with all sections, see nature.com/documents/nr-reporting-summary-flat.pdf

Life sciences study design

All studies must disclose on these points even when the disclosure is negative.

Sample size	Sample size was chosen to have at least three independent measurements of three independent biological replicates for all major experiments. Regarding the multitude of strains, different experimental conditions and different analyses, we consider this size of replicates to be reasonable in terms of scientific output and time. When more tiny differences needed to be quantified (changes in cross section), we extended the analyses to five to six biological replicates.
Data exclusions	Sample size was chosen to have at least three independent measurements of three independent biological replicates for all major experiments. Regarding the multitude of strains, different experimental conditions and different analyses, we consider this size of replicates to be reasonable in terms of scientific output and time. When tinier differences needed to be quantified (changes in cross section), we extended the analyses to five to six biological replicates.
Replication	All attempts were successful and hence are given as experimental data in the manuscript, except the few experiments of the long term PAM recording indicated above.
Randomization	This is not relevant to our study.
Blinding	Blinding was not really relevant to our study. However, for the different strains described in the manuscript, codes were used. When we performed the first cross section and fluorescence lifetime experiments, the principal experimental investigator of these experiments did not know the meaning of these codes and hence evaluated the data without knowing at all what to expect for which strain.

Reporting for specific materials, systems and methods

We require information from authors about some types of materials, experimental systems and methods used in many studies. Here, indicate whether each material, system or method listed is relevant to your study. If you are not sure if a list item applies to your research, read the appropriate section before selecting a response.

Materials & experimental systems

n/a	Involved in the study	
☐	☒	Antibodies
☒	☐	Eukaryotic cell lines
☒	☐	Palaeontology
☒	☐	Animals and other organisms
☒	☐	Human research participants
☒	☐	Clinical data

Methods

n/a	Involved in the study	
☒	☐	ChIP-seq
☒	☐	Flow cytometry
☒	☐	MRI-based neuroimaging

Antibodies

Antibodies used	Antibodies used in this study are indicated specifically in the manuscript. All of them are provided by Agrisera (Vannas Sweden). The commercially available Rubisco antibody can be bought (AS03 037), the Lhcx AB was designed by us and produced by Agrisera. It can be obtained from us upon request.
Validation	The Rubisco antibody is indicated to recognize Rubisco of P. tricornutum (website Agrisera). The polyclonal Lhcx antibody was raised in rabbits against a specific peptide which is present in the C-terminus of all 4 Lhcx proteins in P. tricornutum (indicated in the manuscript). It has been verified via ELISA by Agrisera by comparing immune serum versus pre-immune serum.
